# Combining school-catchment area models with geostatistical models for analysing school survey data from low-resource settings: Inferential benefits and limitations

**DOI:** 10.1016/j.spasta.2022.100679

**Published:** 2022-06-29

**Authors:** Peter M. Macharia, Nicolas Ray, Caroline W. Gitonga, Robert W. Snow, Emanuele Giorgi

**Affiliations:** a Centre for Health Informatics, Computing, and Statistics, Lancaster Medical School, Lancaster University, Lancaster, LA1 4YW, UK; b Population Health Unit, Kenya Medical Research Institute-Wellcome Trust Research Programme, PO, Box 43640, Nairobi, Kenya; c GeoHealth group, Institute of Global Health, University of Geneva, Geneva, Switzerland; d Institute for Environmental Sciences, University of Geneva, Geneva, Switzerland; e Centre for Tropical Medicine and Global Health, Nuffield Department of Medicine, University of Oxford, Oxford, OX3 7LG, UK

**Keywords:** Catchment area models, Disease mapping, School survey, Missing locations, Model-based geostatistics, Prevalence

## Abstract

School-based sampling has been used to inform targeted responses for malaria and neglected tropical diseases. Standard geostatistical methods for mapping disease prevalence use the school location to model spatial correlation, which is questionable since exposure to the disease is more likely to occur in the residential location. In this paper, we propose to overcome the limitations of standard geostatistical methods by introducing a modelling framework that accounts for the uncertainty in the location of the residence of the students. By using cost distance and cost allocation models to define spatial accessibility and in absence of any information on the travel mode of students to school, we consider three school catchment area models that assume walking only, walking and bicycling and, walking and motorized transport. We illustrate the use of this approach using two case studies of malaria in Kenya and compare it with the standard approach that uses the school locations to build geostatistical models. We argue that the proposed modelling framework presents several inferential benefits, such as the ability to combine data from multiple surveys some of which may also record the residence location, and to deal with ecological bias when estimating the effects of malaria risk factors. However, our results show that invalid assumptions on the modes of travel to school can worsen the predictive performance of geostatistical models. Future research in this area should focus on collecting information on the modes of transportation to school which can then be used to better parametrize the catchment area models.

## 1 Background

In low resource settings, the prevalence of parasitic infections are important indicators to guide control. Surveys are traditionally undertaken among sampled residents in communities or from fixed locations that serve these communities, such as schools. School-based sampling has been used for decades in Sub Saharan Africa (SSA) to inform the targeted responses for helminth (Hodges et al., 2011; Tchuem Tchuenté et al., 2012; Soares Magalhães et al., 2011), schistosomiasis (Hodges et al., 2011; Tchuem Tchuenté et al., 2012; Soares Magalhães et al., 2011; Knowles et al., 2017; Fornace et al., 2020; Clements et al., 2006) and malaria (Gitonga et al., 2010; Brooker et al., 2009; Ashton et al., 2015; Mathanga et al., 2015) control. School-based surveys for parasitic diseases represent convenient and cost-effective sampling strategies to provide local disease information, where school attendance is high, and infections can be asymptomatic (Ashton et al., 2011; Brooker et al., 2009; Drake et al., 2011; Mathanga et al., 2015; Stevenson et al., 2013; Takem et al., 2013). Sample school surveys can be powered to provide estimates of malaria prevalence at geographical units (e.g., districts) used for decision making. For example, targeting certain districts with high malaria prevalence. More commonly, the lack of statistical power from minimally sampled schools has involved the applications of model-based geostatistical (MBG) methods (Diggle et al., 1998), using aggregated information at sampled school locations to provide information at unsampled locations (Soares Magalhães et al., 2011; Fornace et al., 2020; Clements et al., 2006; Ashton et al., 2011).

MBG methods for disease mapping have become an established set of modern and robust statistical tools (Diggle et al., 1998) that are used to inform disease control strategies (Macharia et al., 2018; Biggeri and Catelan, 2012), especially in low-resource settings where disease registries are non existent or incomplete (Alegana et al., 2020; Pop et al., 2019; Stefan et al., 2014). Typically, MBG for disease prevalence mapping aim to predict a disease risk surface using data consisting of a finite set of locations *x_i_*, for *i* = 1,…, *N*, where a number of *n_i_* individuals are tested for a disease of interest and of which *y_i_* test positive. Ideally, the *x_i_* would correspond to the locations where individuals contracted the disease but, in practice, this is often difficult, if not impossible, to assess and access. In most geostatistical analyses of epidemiological data, the location of the school or village is used as the main location of exposure to the disease of interest.

However, in most school malaria surveys, due to resource constraints, precise spatial information on the residence of the children is rarely collected. Only the geographical location of the school is collected, hence the uncertainty in the household location of the children within a school catchment area. Consequently, when mapping malaria prevalence using school survey data, the school location is often used as the exposure location due to missing spatial information on the residential location. For example, in Ashton et al. (2015), Binomial geostatistical models are fitted to serological indicators collected from school surveys, while using the location of the school to define the spatial correlation between observations. The same approach has been used in other studies that have combined school-based data with community-based data (Macharia et al., 2018; Runge et al., 2020). In Stensgaard et al. (2011), the problem of the use of the school locations to estimate covariates effects on prevalence is alleviated by averaging the covariates within 1 km around each school location. However, this approach is questionable, since it implicitly assumes a circular school catchment area with a radius of 1 km while still making use of the school location in the computation of the spatial correlation.

A common problem to these approaches is that, by allocating individuals to locations that are less representative of their actual exposure to malaria, it can potentially bias the spatial structure of the MBG model and thus invalidate the predictive inferences on prevalence. This is especially important for school going children since a bite from an infected *Anopheles* mosquito is more likely to occur during night, when children are usually at home (Maxwell et al., 1998).

The statistical problem addressed in this paper is related to the problem that arises in passive surveillance when dealing with spatially aggregated disease counts; see for example Cameron et al. (2021). In Sturrock et al. (2014), aggregated case data reported at health facility are modelled by defining the probability of observing a case at any given location as the product of being a case and the probability that individual would seek treatment. However, in this work the random effects that modulate the probability of being a case are modelled as a spatially discrete process that is tied to the specific definition of the hospital catchment areas. The fine scale predictions of prevalence in this case, are thus a product of spatially continuous covariates and spatially discrete random effects. In this paper, we overcome this limitation by adopting a spatially continuous random effect whose properties are independent of the catchment areas. However, one key difference between the problem addressed in this paper and previous work on aggregated counts at health facilities, is that the latter problem is more naturally addressed using spatial point patterns methods. In other words, if locations were fully observed, a Log-Gaussian Cox process (LGCP) would be a natural modelling option to model reported cases at health facilities, whilst in our case, the location is not the object of interest, but rather the prevalence associated with that. Diggle et al. (2013), using LGCPs, provide a principled solution for fine-scale mapping by modelling the spatially aggregated disease counts as the realization of an aggregated spatially continuous stochastic process. As in Cameron et al. (2021), the methods that have been developed in the context of spatially aggregated passive surveillance data are based on log-linear models do not provide a solution to the problem addressed in this paper. Nelli et al. (2020) develop methods to combine passive and active surveillance data, however the sampled locations of the latter are assumed to be observed.

To the best of our knowledge, no statistically rigorous solution has been proposed to handle the problem of missing residence locations from malaria school survey data in a geostatistical model. In this paper, we provide a first solution to this problem and compare the predictive inferences on prevalence resulting from this novel approach with standard statistical approaches that use the school location to model the spatial correlation.

## 2 Methods: combining school catchment area models with geostatistical models

In this study, the set of residence locations *X*, unlike in standard geostatistical analyses, must be treated as a random variable and a suitable distribution for this must first be defined. Once we have defined the statistical model for *X*, we can use this to impute likely values for the unobserved residence locations of children and incorporate these into a geostatistical model for disease prevalence. This process allows us to rigorously acknowledge the uncertainty arising from the missing residence locations in the predictive inferences of disease prevalence. However, this begs the two following questions: (1) what is a suitable model for *X*? (2) How to combine the model for *X* with a geostatistical model for disease prevalence?

To answer these questions, our approach is to develop a marked Poisson process for the unobserved residence locations, *X*, with marks corresponding to each of the schools, and whose domain is restricted by a school catchment area (SCA) model informed by several factors that affect travel (road network, land use, protected areas, water bodies and travel speed). We then use the resulting model for *X* to generate samples of locations and feed these into a MBG model for disease prevalence. This approach presents several computational issues which we address using an stochastic partial differential equation (SPDE) approximation (Lindgren et al., 2011) for spatial Gaussian processes.

We first introduce the framework for accounting for missing residence locations in the context of disease prevalence mapping which entails creating SCAs and generating samples of the most likely residential locations *X*. This is then followed by the estimation of the model parameters and spatial prediction of disease prevalence within a predefined geographical area of interest. The application of the proposed modelling framework is illustrated through two case studies of malaria mapping in Western Kenya using school survey data.

### 2.1 Accounting for missing residence locations in a geostatistical model for disease prevalence mapping

In this section, we formalize and provide a solution to the problem of how to propagate the uncertainty in geostatistical models for prevalence mapping, arising from the lack of residence locations in school survey data.

Let [·] be a shorthand notation for “the density function of the random variable ·”. We then use *X* = {*X*_1_,…, *X_n_*} to denote the random variables representing the set of unobserved residence locations, and *Y* = (*Y*_1_,…, *Y_n_*) for the observed individual-level binary outcomes indicating a positive (*Y_i_* = 1) or negative (*Y_i_* = 0) test. Finally, let *S* = {*S*(*x*) : *x* ∈ ℝ^2^} be an isotropic and stationary Gaussian process with mean zero and covariance function

$$\mathrm{cov}\left\{S(x),S\left(x^{\prime }\right)\right\}=\sigma^{2}\rho (u),u=∥x-x^{\prime }∥.$$

Assuming that *X* and *S* are independent of each other, or in other words assuming a non-preferential sampling design, the joint distribution of *X*, *S* and *Y* can be written as

(1) [X,S,Y]=[X][S][Y∣S,X]=[X][S][Y∣S(X)]

where *S*(*X*) = {*S*(*x*) : *x ∈ X*} and [*Y*|*S*(*X*)] is a set of mutually independent Bernoulli variables, i.e.

$$[Y|S(X)]=\prod_{i=1}^{n}\left[Y_{i}|S\left(X_{i}\right)\right].$$

When residence locations *X* are observed, then [*X*] is irrelevant for drawing inferences on *S*, and thus can be ignored. In our case, instead, because of the missingness of *X*, the distribution [*X*] must be integrated out from the likelihood function for the unknown vector of parameters *θ*, i.e.

(2) $$L(\theta )=[Y;\theta ]=\int_{A^{2n}}\int_{{\mathbb{R}}^{n}}[X][S][Y|S(X)]\;dS\;dX.$$

where *A* ⊂ ℝ^2^ is our geographical region of interest.

To estimate *θ* from (2) and draw predictive inferences on *S*, we then first need to specify a model for the location process [*X*].

### 2.2 Modelling [X] using school catchment area models

To model the location process [*X*], we propose to generate a school catchment area (SCA) based on travel time from the residence of children. The SCA is then used as the boundary of the area from which we shall draw samples of the residence locations using population density information. More specifically, an SCA is defined as the geographical area or zone around a school that draws majority of the students (Macharia et al., 2021b). One approach is to use so-called *gravity models* to express the decreasing likelihood of geographically accessing a school, as the distance or travel time to that school increase (Guagliardo, 2004) and they have been used to model catchment areas for healthcare planning (Macharia et al., 2017; Alegana et al., 2012; Guagliardo, 2004). Hence, *spatial accessibility* of a residence location *x* is defined as

(3) $$a(x)=\underset{j}{\sum^{\text{​}}}\frac{c_{j}}{f{\left(x,x_{j}\right)}^{\gamma }},$$

where: *c_j_* is a constant that expresses the capacity of a school placed at location *x_j_*, and can, for example, be quantified by the number of teachers in that school; *f* (*x*, *x_j_*) is the impedance (traveltime) between locations *x* and *x_j_*; *γ* is a gravity decay coefficient, also referred to as the travel friction coefficient.

Real SCAs vary in size according to the underlying population distribution, number of schools in the surrounding area, school capacity and other school *attractiveness* factors. School attendance and geocoded residential location *X*, when available, can be used to estimate the parameters in (3). However, in the absence of such data, as in the scenario considered in this paper, a feasible and useful alternative is to use cost distance to define the spatial accessibility function *a*(*x*) and use optimization algorithms that allow to identify an optimal route of travel to school. Based on this definition of spatial accessibility, an SCA is then defined as the geographical area encompassing all locations closest, in terms of travel time, to that school than any other school. The travel time to school is dependent on the speed of the mode of travel to school which, in turn, is affected by several spatial layers, including the road network that students travel on, land cover layer to represent the travel impedance in spaces between the roads, and barriers to movement comprising water bodies, flooded areas, and protected areas. Barriers are considered impassable, except in the presence of a bridge where a road intersects a barrier such as a river. To model the speed of the mode of transport and identify the optimal route to school, we propose to use terrain-based least-cost path distance calculation (Ray and Ebener, 2008) and geospatial layers representing factors that affect travel to generate a travel time grid indicating the time taken by a student to travel from their residential location to the nearest school.

Each road class and land cover are assigned a mode of transport and travel speed, which ideally are informed by observational data of schools attendance behaviour in a specific region. However, in low resource settings, such data are rarely available (Macharia et al., 2021b) and a common alternative is the review of literature in similar settings to assemble road speeds and modes of transport in one or more travel scenarios. We provide details on the parametrization of the speed functions for our study regions in Kenya, in Section 3.2.

After generating a SCA, we spatially link this with a population density raster (Stevens et al., 2015) which is then used to sample residential locations within the SCA. The population density rasters are constructed through dasymetric techniques that redistributes national census population counts from administrative units to high spatial resolution (e.g. 100 metres m × 100 metres) (Mennis, 2009; Stevens et al., 2015). Let *C* denote the area encompassed by the boundaries of a given SCA; we then generate samples *X_(j)_* = {*x*_1(*j*)_,…, *x*_*n*(*j*)_}, for *j* = 1,…, *B* for the unobserved residence locations, *X*, by using a fine regular grid covering *C* to approximate the probability of sampling a location *x* ∈ *c*, given by

$$\frac{\lambda (x)}{\int_{C}\lambda (u)du},$$

where *λ*(*x*) denotes the population density at *x*.

### 2.3 Approximating [S] and the likelihood function: from parameter estimation to spatial prediction

The resulting Monte Carlo samples *X*_(*j*)_, *j* = 1,…, *B*, obtained as described in the previous section, are now used to approximate (2) as

(4) $$L(\theta )\approx \int_{{\mathbb{R}}^{n}}\frac{1}{B}\sum_{j=1}^{B}[S][Y|S\left(X_{\left(j\right)}\right)]\;dS.$$

To avoid the computation of *B* covariance matrices for each of the stimulated samples *X*_(*j*)_, we approximate *S* using a piece-wise linear approximation for *S*(*x*). More specifically, we partition the study region *A* into a set of non-intersecting triangles that share at most a common edge. For the generation of the triangles we follow the approach outline in Section 2.2.2 of Krainski et al. (2018).

We then approximate the spatial Gaussian process as

(5) $$S(x)=\sum_{k=1}^{m}b_{k}(x)W_{k}$$

where *b_k_*(*x*) are basis functions and the *W_k_*, for *k* = 1,… *m* are Gaussian random variables. Based on the created mesh, we then define the basis functions *b_k_*(*x*) using barycentric coordinates, in which the location of a point is specified by reference to vertices of a triangle. It then follows that *b_k_*(*x*) takes a non-zero value whenever a point *x* falls inside a triangle identified by the vertex associated with *b_k_*(*x*) such that $\sum_{k=1}^{m}b_{k}(x)=1$; for more details see Section 2.2.2 of Krainski et al. (2018). Following Lindgren et al. (2011), we assume that *W* = (*W*_1_,…, *W_m_*) follows a zero-mean multivariate Gaussian distribution with precision matrix *Q*, which is chosen so as to approximate a Matérn spatial field with smoothness parameter *κ* = 1 and scale parameter *ϕ*. Hence, we write

$$Q={\left(\frac{\phi^{2\kappa }\text{Γ}(\kappa )}{4\pi \sigma^{2}\text{Γ}(\kappa +2)}\right)}^{2}\left(\phi^{-4}C+2\phi^{-2}G_{1}+G_{2}\right)$$

where *C*, *G*_1_ and *G*_2_ are sparse matrices whose entries are non-zero only for pairs of vertices that share the same triangles; for more details on how the entries of *C*, *G*_1_ and *G*_2_ are defined, we refer the reader to Lindgren et al. (2011).

Let *x*_*i*(*j*)_ denote the *i*th element of *X*_(*j*)_ for *i* = · · ·, *n*; following from (5), we then define [*Y*|*W*, *X*_(*j*)_] as a set of mutually independent Bernoulli variables with linear predictor

(6) $$\log\left\{\frac{p\left(x_{i(j)}\right)}{1-p\left(x_{i(j)}\right)}\right\}=d^{\text{⊤}}(x_{i(j)})\beta +\sum_{k=1}^{m}b_{k}\left(x_{i(j)}\right)W_{k}$$

where *d*(*x*_(*j*)_) is a vector of spatial covariates, recorded at location *x*_(*j*)_, with associated regression coefficients *β*.

Let us split the vector of unknown parameters *θ*, into covariance parameters *ψ* = (*σ*^2^, *ϕ*) of the spatial process and regression coefficients *β*, and reparametrize (4) based on *W* to give

(7) $$L(\theta )\approx \int_{{\mathbb{R}}^{m}}[W;\psi ]\left(\frac{1}{B}\sum_{j=1}^{B}\left[Y|W,X_{\left(j\right)};\beta \right]\right)\;dW.$$

Since the above integral is intractable, we use Monte Carlo methods to approximate the likelihood function as follows. Let *ψ*_0_ and *β*_0_ be our initial guesses for the parameters *ψ* and *β*, respectively. To simplify the notation let $g(Y,W;\beta )=\sum_{j=1}^{B}[Y|W,X_{\left(j\right)}]/B$; we then rewrite (7) as

(8) $$\begin{array}{ll} L(\theta )\;\approx & \int_{{\mathbb{R}}^{m}}[W;\psi ]g(Y,W;\beta )dW\\ =\int_{{\mathbb{R}}^{m}}[W;\psi ]g(Y,W;\beta )\frac{\left[Y,W;\psi_{0},\beta_{0}\right]}{\left[Y,W;\psi_{0},\beta_{0}\right]}dW\\ \propto \int_{{\mathbb{R}}^{m}}\frac{\left[W;\psi ]g(Y,W;\beta \right)}{[W;\psi_{0}]g\left(Y,W;\beta_{0}\right)}[W|Y;\psi_{0},\beta_{0}]dW\\ =E_{0}\left[\frac{\left[W;\psi ]g(Y,W;\beta \right)}{\left[W;\psi_{0}]g(Y,W;\beta_{0}\right)}\right] \end{array}$$

where *E*_0_ is the expectation taken with respect to the distribution of *W*, conditional on *Y*, with parameters *ψ*_0_ and *β*_0_ or, using the shorthand notation, [*W*|*Y*; *β*_0_, *ψ*_0_]. Hence, we approximate (7), by sampling from [*W*|*Y*; *β*_0_, *ψ*_0_] using a Metropolis Hastings independence sampler with proposal distribution given by a multivariate Gaussian distribution with mean and covariance matrix corresponding to the mode and inverse of the negative Hessian of [*W*; *ψ*_0_]*g*(*Y*, *W;* *β*_0_), respectively.

When the goal of the analysis is primarily focused on spatial prediction and not on drawing inferences on *β*, the computational burden can be alleviated by first estimating *β* using a simpler model that ignores spatial correlation, which is obtained by setting *S*(*x*) = 0 for all *x* to give $\log\{\tilde{p}(x_{\left(j\right)})/(1-\tilde{p}(x_{\left(j\right)}))\}=d^{\text{T}}\left(x_{\left(j\right)}\right)\beta$. The likelihood function of this non-spatial model is given by

$$\tilde{L}(\beta )=\frac{1}{B}\sum_{j=1}^{B}\prod_{i=1}^{n}\tilde{p}(x_{i(j)})[1-\tilde{p}(x_{i(j)})]$$.

After maximizing the above function with respect to *β*, we obtain $\tilde{\beta }$ which we now plug-in into the linear predictor (6). This then leads to a simplified likelihood function for the covariance parameters *ψ*, expressed by

(9) $$L_{\tilde{\beta }}(\psi )=E_{0}\left[\frac{\left[W;\psi ]g(Y,W;\tilde{\beta }\right)}{\left[W;\psi_{0}\right]g(Y,W;\tilde{\beta })}\right]=E_{0}\left[\frac{\left[W;\psi \right]}{\left[W;\psi_{0}\right]}\right]$$

By maximizing the above function, we finally obtain $\tilde{\phi }$ as our point estimate of *ψ*. Since the primary objective of our case study in predicting malaria prevalence, we adopt this approach in our two applications.

Spatial prediction of prevalence is carried by plugging the point estimates $\tilde{\beta }$ and $\tilde{\phi }$ into (6).

## 3 Application 1: large scale mapping of malaria prevalence across eight counties of Western Kenya

### 3.1 Data

**Fig. 1:**
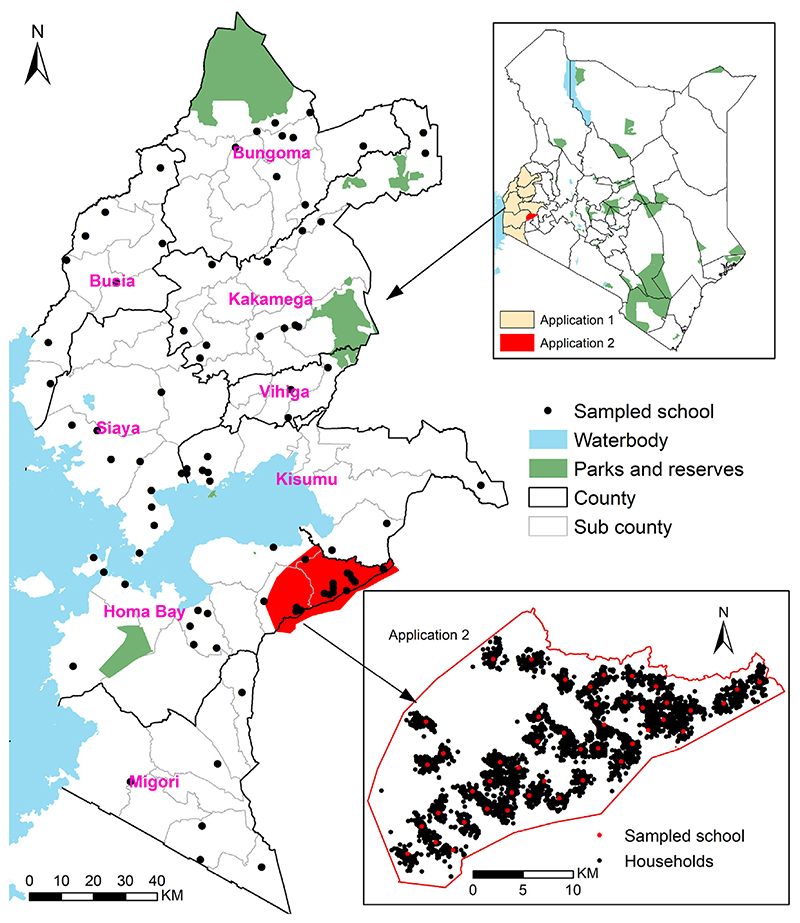
Study areas of the applications of Sections 3 and 4, both located in Western Kenya. Solid black (counties) and light grey (sub-counties) lines represent administrative units. The map in the lower right corner shows the study area and sampled households from the application of Section 4.

We analyse data from a national school-based survey of malaria prevalence conducted in 2009 in Kenya; full details of the survey are provided elsewhere (Gitonga et al., 2010). Here, we consider 84 sampled public day primary schools located in a high malaria transmission region of Western Kenya, covering eight counties and 62 sub-counties close to Lake Victoria. All eight counties had at least a school surveyed while 50 sub-counties (81%) had at least a surveyed school (Fig. 1). At each school approximately 100 children aged 4–22 years were randomly sampled from classes 2–6 for a total of 9103 children. The majority of the children (91%) were aged between 8 and 14 years while only 0.5% were aged at least 17 years. Each sampled child provided a finger-prick blood sample that was used to detect Histadine Rich Protein (HRP) as evidence of recent *Plasmodium falciparum* infection using a rapid diagnostic test (RDT) (Paracheck-Pf device). Slides from all RDT positive samples were examined using light microscopy and 10% of all RDT negatives. A child was deemed positive for malaria when parasites were detected on microscopy. The location of the school was recorded using a hand-held Global Positioning System (GPS) device (Gitonga et al., 2010).

In addition to the malaria school survey data, a set of spatial covariates were used to assist the spatial prediction of prevalence at unsampled locations. These are listed and described in Table 1. Before including these covariates in our model, we explored the association of each of the covariates listed in Table 1 by taking the *empirical logit transformation* (Stanton and Diggle, 2013) of the total number of cases recorded at each school and plotted this against each of the covariates, whose value on the x-axis was obtained by taking its average within the SCAs.

### 3.2 Model specification for [X]

We first assembled a list of all public day primary schools in 2009 (Mulaku and Nyadimo, 2011) (Figure S1.1 in *Supplementary material 1*) and variables representing factors that are known to affect travel (Table 2 and Figures S1.2 to S1.4 in *Supplementary material 1*) in order to define the speed of a specific mode of travel at any location *x* of the study area (Fig. 1). To compute travel time, we used AccessMod (version: 5.7.3-alpha) (Ray and Ebener, 2008), an open-source package that models geographical access using terrain-based least-cost path distance calculation (Ray and Ebener, 2008). We used the ‘merge land cover’ module of AccessMod to merge the land cover, road network, water bodies, national parks and reserves, and obtain a ‘merged landcover’ raster at 100-metre resolution. Travel speeds were then assigned to each land cover type and road class.

School going children can use different transport modes to reach their school. Since data on modes of travel were not available, we considered three different models corresponding to travel scenarios based on different assumptions for the means of transportation used by the children to reach their school.

- *Model W.* It assumes that all students walk to school with speeds ranging between 5 km/h to 0 km/h (Samimi and Ermagun, 2013; Macharia et al., 2017; Mehdizadeh et al., 2017; Alegana et al., 2021) as detailed in Table 3.
- *Model WB.* Travel to school is assumed to be a combination of walking and bicycling with a maximum speed of 10 km/h for bicycles.
- *Model WM*. Travel to school is assumed to be combination of walking and motorized transport (motorcycles, private and public vehicles). The maximum motorized speed was 50 km/h.

Table 3 shows how velocities vary in each transport scenario for different types of terrains.

To generate SCAs for all schools in the region, we used the “accessibility” module of AccessMod to run an anisotropic travel time computation for each of three aforementioned travel models. The anisotropic option considers the slopes derived from the Digital Elevation Model (DEM) (Table 2) and travelling towards the school (direction of travel) to correct for walking (10) and bicycling speeds (Ray and Ebener, 2008; Tobler, 1993). Hence, we express the adjusted walking speed *W_v_* as

(10) $$w_{v}=w_{f}\times e^{\exp\{-3.5|k+0.05|\}}$$

where *w_f_* is the speed on flat surface on the landcover considered and *k* is the slope derived from the elevation.

**Fig. 2:**
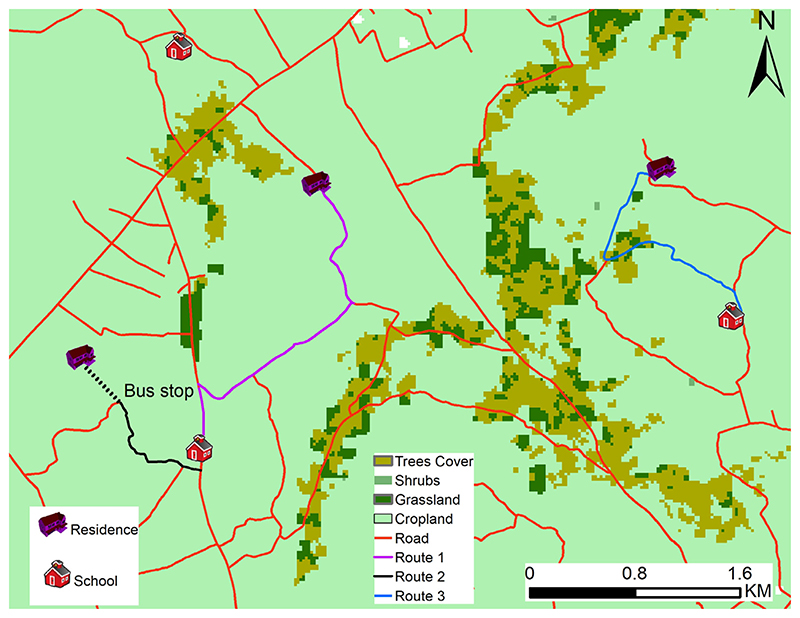
A map showing examples of optimal routes taken by students, under different mode of travel. For a detailed explanation, we refer the reader to the main text.

The adjustment for the walking mode of travel decreases walking speed as the slope increases, while increasing the walking speed for a negative slope, according to the Tobler’s hiking function (Tobler, 1993). Bicycling speed are adjusted assuming that the increased speed on negative slopes does not exceed twice the speed on flat ground (Ray and Ebener, 2008). The motorized speeds were not adjusted for the slope as vehicles are powered by an engine. Fig. 2 provides three example routes that help to illustrate how the chosen parametrizations for the different speed affect travel time. Using a regular grid with spatial resolution of 20-m, we estimate that a student riding a bicycle on Route 3 (blue-low class road), would travel at an average speed of 10 km per hour taking a total of 14 min to reach their school. On Route 2, a student will first walk through the area without a road at 5 km per hour (6 min), take a vehicle at the bus stop travelling at a speed of 50 km/h (2 min), taking a total of 8 min to reach their school. Finally, a student taking Route 1 (purple — high road class) would take 4 min using a motorized vehicle at 50 km per hour. The routes are least cost paths determined by the cost distance algorithm with speed adjusted as described above.

We used the ‘cost allocation’ option in AccessMod to compute the cost allocation grid delineating all SCAs. The cost allocation algorithm is similar to that used for a Voronoi diagram in Euclidean distance analysis, or to a location–allocation model in a network analysis (Ouma et al., 2021). The generated SCAs for the 84 sampled schools were then spatially linked to the 2009 population density maps (Stevens et al., 2015) and 10,000 samples for the residence locations, *X*, of the children were generated. The choice of least cost distance and cost allocation algorithms to model travel time and SCAs, respectively was appropriate given they account for transport factors (Table 2) and corresponding speeds (Table 3). This is unlike the use of straight line distances, Voronoi diagram and location–allocation model that do not account for these travel factors. See Ouma et al. (2021) and Macharia et al. (2021b) for further details.

Finally, three geostatistical models under the different travel scenarios described above were fitted; the mesh used to define the piece-wise linear approximation of the spatial Gaussian process is shown in Figure S1.12 of *Supplementary material 1.* In addition to these models, we also fit two geostatistical models that use the school location to model the spatial correlation in the data but make different use of the covariates: the SL model uses the value of the covariate at the location of the school; the SCLA model uses the averaged covariate within SCAs.

### 3.3 Simulation study

We carry out a simulation study to pursue two objectives:

1. to quantify the inferential benefit of accounting for the uncertainty in the location of residence;
2. to understand how the mis-specification of the mode of travel may affect the predictive inferences for prevalence.

To this end, we then proceed through the following iterative steps.

Step 1. Generate a data-set of binary outcomes indicating the malaria status of children, under the geostatistical model that assumes that children use “walking” only (Model W) as a mode of travel to school.

Step 2. Fit five statistical models to the simulated data-set from the previous step: model W (the true model), model WB, model WM, model SL and model SLCA.

Step 3. Generate prevalence predictions $\hat{p}$ and exceedance probabilities *e*(*x*) for a 30% prevalence threshold, over a regular grid covering the study area, for each of the five models.

Step 4. Using the exceedance probabilities *e*(*x*) from the previous step, classify each pixel as being above 30% if *e*(*x*) > *l* and below 30% if *e*(*x*) < *l*, where *l* is obtained by maximizing the specificity and sensitivity of the classification.

Step 5. Repeat Step 1 to Step 4, 1000 times.

Let *p*(*x*) denote the true prevalence. Using a regular grid $\left(x_{1}^{*},\ldots ,x_{q}^{*}\right)$ at 10-metre resolution, we then summarize the predictive performance using the following indices.

- Average bias: $\sum_{j=1}^{q}(\hat{p}(x_{j}^{*})-p(x_{j}^{*}))/q$
- Root-mean-square prediction error: $[\sum_{j=1}^{q}{\left(\hat{p}(x_{j}^{*})-p(x_{j}^{*})\right)}^{2}/q{]}^{1/2}$
- Sensitivity: the average proportion across all simulations of pixels that are correctly classified as exceeding 30% prevalence based on the classification in Step 4.
- Specificity: the average proportion across all simulations of pixels that are correctly classified as non-exceeding 30% prevalence based on the classification in Step 4.

### 3.4 Results

**Fig. 3:**
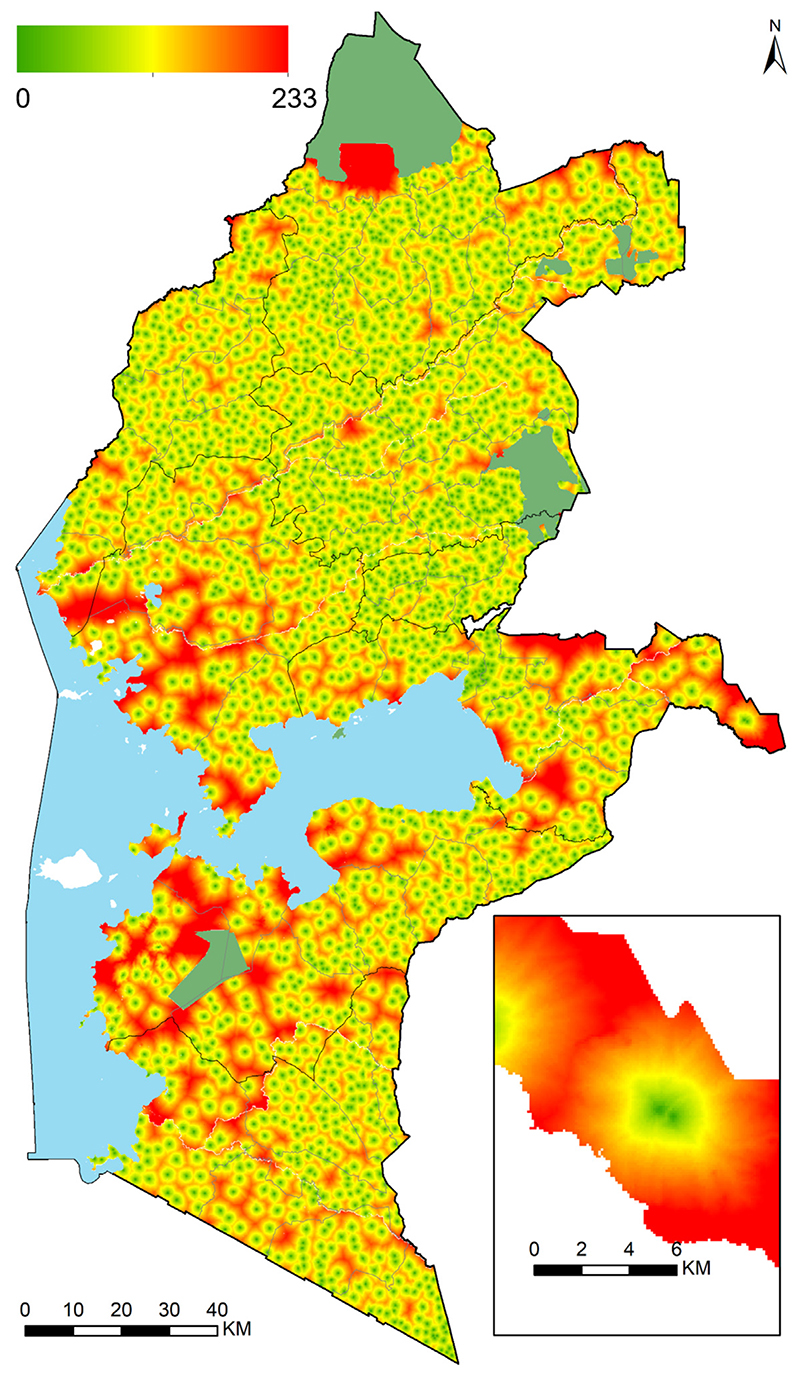
Travel time (in minutes) to the nearest public day primary school for all 2170 public primary schools in Western Kenya in 2009 ranging from 0 min (light green) to 233 min (red) *Model W*. Results of *Model WB* and *Model WM* are shown in Supplementary material 1.

Geographic accessibility (travel time in minutes) to the nearest public day primary school in Western Kenya was highly heterogeneous in 2009. The combination of walking and motorized transport (*Model WM*) provided the fastest option of reaching the nearest school (ranging 0 to 128 min) (see Figure S1.7 in *Supplementary material 1*) while walking-only scenario (*Model W*) ranged between 0 and 233 min (Fig. 3). Walking and bicycling combined (*Model WB*) ranged between 0 and 203 min (see Figure S1.6 in *Supplementary material 1*). The higher travel times were common in areas adjacent to the mountains and at county border regions. Overall, the majority of the school going children had good geographic access to their nearest primary schools in 2009. Across the eight counties, 68.4%, 74.1% and 78.3% of all the school going children (2.2 million) in the region in 2009 were within 30 min of the nearest primary school for models W, WB and WM, respectively.

A universal gold standard (threshold) of travel time/distance to the nearest primary school does exist due to differences in population distribution, context, geography, infrastructure, and resources between countries. In Kenya, The Ministry of Education aims to have a school within 2 km walking distance of every household which is about 24 min based on an average walking speed of 5 km/h. The average travel time (distance) in the region was 28 min (2.33 km) while 65% of the school-going children were within 24 min. The western Kenya average is fairly comparable to Rwanda (1.7 km), higher than Gutemala (1.1 km), South Africa (1.1 km) and Peru (1.4 km) but lower than Tanzania (5.9 km) (Rodriguez-Segura and Kim, 2021). However, these estimates should be interpreted with caution given differences in methods, input data and context.

**Fig. 4:**
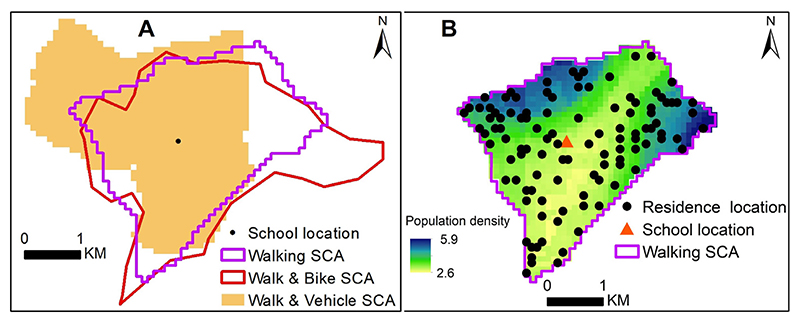
A: Sample of school catchment areas generated from each of transport scenarios; walking *(Model W*), walking and bicycling *(Model WB)* and walking and motorized *(Model WM*). The school is shown as a black dot; B sampled locations for a single iteration from one of the catchment areas *(Model WB*) overlaid on a population distribution map (Stevens et al., 2015) (green to blue shades). In B, the school is shown as an orange triangle. All the school catchment areas are shown in Supplementary material 1.

The travel time was used as the basis for generating SCAs for each of the three likely travel scenarios to a school. The catchment areas were generated for all the 2170 schools in Western Kenya in 2009 to account for competition of the neighbouring schools for the sampled schools. A subset comprising the 84 sampled schools was then retained for the subsequent analysis. The results of the catchment areas are shown in Figures S1.8 to S1.10 of *Supplementary material 1*. Each school catchment area covered all areas nearer to it (based on travel time) than any other school based on the cost allocation algorithm. This meant that all areas and school going children were covered by their nearest school. The size and shape of the catchment areas were variable within and between models. However, neither of the models provided a systematically smaller or bigger sized catchment area relative to the other catchments from alternative travel scenarios. Fig. 4A shows school catchment areas generated using the three travel scenarios (*Models W, WB and WM*) for a Nasianda Primary School where 108 students were surveyed. Fig. 4B shows generated residential locations, *X*, for one iteration for each child sampled from Nasianda Primary school, based on population density as the intensity of a inhomogeneous Poisson process.

From the explanatory analysis, enhanced vegetation index was excluded from the analysis, since this was found to be highly correlated with both the precipitation and the temperature. The three remaining covariates showed an approximately linear relationship with the empirical logit (see Figure S1.11 in *Supplementary material 1*). Consequently, temperature, precipitation and night time lights were used as spatial predictors for all the five models considered (*Models SL, SLCA, W, WB and WM*) for school going children in Western Kenya.

**Fig. 5:**
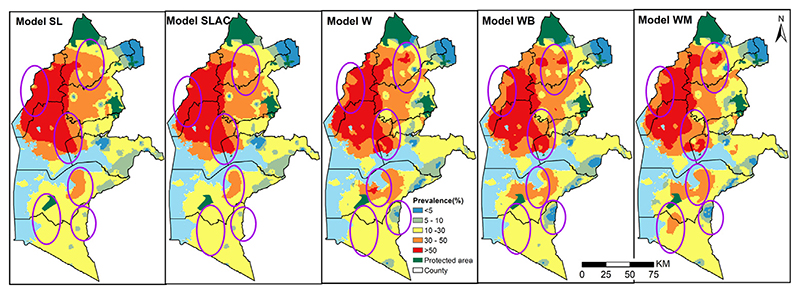
Annual predicted mean malaria prevalence at 1 × 1 km spatial resolution ranging from 0% (blue) to 77.5% (dark red) in Western Kenya in 2009 for 2 standard statistical models *(SL and SLCA)* and three models accounting for locations uncertainty *W, WB, WM*. Ellipses show areas with differences. The protected areas are shown in green.

All Five models considered provide a similar spatial pattern and identify the Western region as an area of high malaria prevalence in 2009. The north-Western region had the highest predicted prevalence (over 50%), while in the north-east the values range between 30% and 50%. In the Southern region, we find the lowest values of prevalence, ranging approximately between 10% and 30%. Only few small areas showed a predicted prevalence below 10%. Overall across the models, the highest predicted values of prevalence is 77.5% according to model *W* (*X* sampled within SCAs based on walking scenario) followed by *Models WM and WB* with slightly lower values of 75.6% and 75.1%, respectively. The SLCA (school’s location — SCA averaged covariates) model had a maximum predicted prevalence of 72.6% while model SL (school’s location) had 69.15% (Fig. 5).

Based on each of the five models, we also estimated the population of school-going children living in areas having a prevalence of at least 30% in 2009. The SL and SLCA models would have the smallest proportion of about 42.1% and 42.6% respectively. On the other hand, the W, WB and WM models yield similar estimates of 44.3%, 44.6% and 43.5% respectively.

These results suggest that difference between the five models considered can be found in localized areas of the study region. A close inspection of Fig. 5 confirms this, where we used purple ovals to highlight those areas. For example, in Busia county, corresponding to the leftmost ring of Fig. 5), the SL and SLCA models classified areas in the ring as over 50% while the other models predicted the prevalence to be between 30% and 50%

**Fig. 6:**
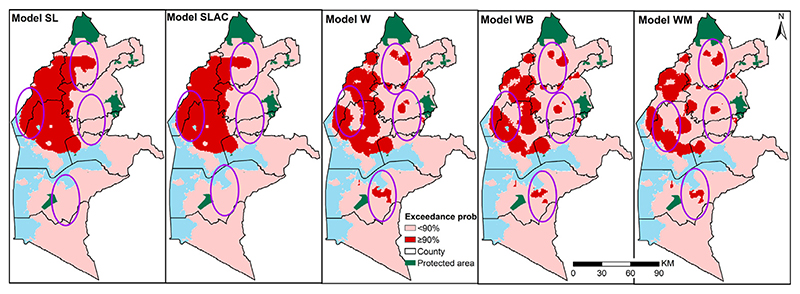
Maps of the predicted exceedance probability for a 30% prevalence threshold with a 90% probability on a 1 by 1 km regular grid in Western Kenya in 2009 for 2 standard statistical models (*SL and SLCA*) and three models accounting for locations uncertainty *W, WB, WM*. Ellipses show areas with differences. The protected areas are shown in green.

In addition to the maps of the predicted prevalence, we also compared the exceedance probabilities (EPs) that malaria prevalence lies above 30%, as shown in Fig. 6. The differences between the five models are more stark in these maps, where we highlighted areas that are at least 90% likely to exceed 30% in red. The extents where malaria prevalence was greater than 30% with over 90% probability was dominant in the north west region. The maps clearly show that the SL and SLCA models identifies a large contiguous areas of EPs larger than 90%, whilst the other three models yield a more heterogeneous pattern in the Northern part of the study region. Unlike the SL and SLCA models, in the Southern part, a relatively large area is shown to have an EP larger than 90% based on W, WB and WM. Approximately 23.1% and 22.6% of the school going children were within the areas where prevalence was classified as over 30% with a probability of at least 90% based on *SL and SLCA models* respectively. On the other hand, *models W, WB, WM* have approximately 19.2%, 16.7% and 14.3%, respectively school going children with the same margins.

The results of the simulation study are presented in Section 3.3. Except for WM model which provides the worst performance, the differences between the other models in terms of the four metrics used, are rather small. These small differences can be explained by the fact that the catchment areas are relatively small to the scale parameter *ϕ*, hence school locations can be used to approximate the residual spatial correlation structure reasonably well in this instance (see Table 4).

## 4 Application 2: Small scale mapping of malaria prevalence in Western Highlands of Kenya

### 4.1 Data

In this section, we analyse data from a school-based survey of malaria prevalence conducted in 2010 in a small area within the region encompassed by the first application as shown in Fig. 1. The full details of the survey are provided elsewhere (Stevenson et al., 2013). The data consists of 46 sampled primary schools, randomly selected from a census of all public primary schools in the area. At each school, 11 boys and 11 girls per class from classes 2 to 6 were selected randomly for a total of 4852 children. Those aged 8–14 and over 17 years were 90% and 1.2% of the total children, respectively. Each sampled child provided a finger-prick blood sample that was used to detect HRP as evidence of recent *Plasmodium falciparum* infection using RDT (Paracheck, Orchid Biomedical Systems, India). The compound of each child sampled at school was located and mapped using a personal digital assistant (PDA) with GPS receiver (Stevenson et al., 2013) and as illustrated in Fig. 1. Important distinction between the first and the second application, is that the latter mapped the compound/households of the school children, the gold standard, while the former did not.

The set of spatial covariates in the first application listed in Table 1 were used. However, because of the low variation of these across the study site of this second application, we carry out the analysis without using any covariates. Factors that affect travel to school are shown in Figure S2.1 (*Supplementary material 2*).

### 4.2 Model specification for [X]

We used the same approaches to estimate travel time and model SCAs as defined in the first application in terms of factors that affect travel (Table 2) and corresponding travel speeds (Table 3) for the three apriori defined travel scenarios (*W, WB and WM).* Likewise, 10,000 sample residence locations, *X*, of the children were generated constrained by the SCAs from the 46 sampled schools.

Five geostatistical models were then fitted. The first three were based on sampled residence locations, *X* using the mesh shown in Figure S2.6 of *Supplementary material* 2. The other two models were based on school location (*model SL*) and household location, the *gold standard* model denoted as *model HL*.

In this application, the HL model, which was not available in the first application, represents our gold-standard reference which we use to discriminate which model delivers the best predictive performance. Hence, we used the following indicators to quantify which model yields a predictive surface for prevalence that follows more closely the HL model.

- Average bias: $\sum_{j=1}^{q}(\hat{p}(x_{j}^{*})-{\hat{p}}_{HL}(x_{j}^{*}))/q$
- Root-mean-square prediction error: $[{\sum_{j=1}^{q}{\left(\hat{p}(x_{j}^{*})-{\hat{p}}_{HL}(x_{j}^{*})\right)}^{2}/q]}^{1/2}$

In the above expressions, ${\hat{p}}_{HL}(x_{j}^{*})$ denotes the predicted prevalence a grid location $x_{1}^{*}$ from the HL model.

### 4.3 Results

Travel time (in minutes) to the nearest primary school and the corresponding SCAs are shown in Figure S2.2 of the *Supplementary material 2*. Travel time to the nearest school was high and heterogeneous across the three models. Almost all the children were within half hour of the nearest primary school in the three travel scenarios. Specifically, 97.6%, 98.3% and 97.8% of all school going children in the area (347,013) were within half-hour threshold for models *W, WB and WM*, respectively. Those outside the threshold were adjacent to a river and on the edge of the study area.

**Fig. 7:**
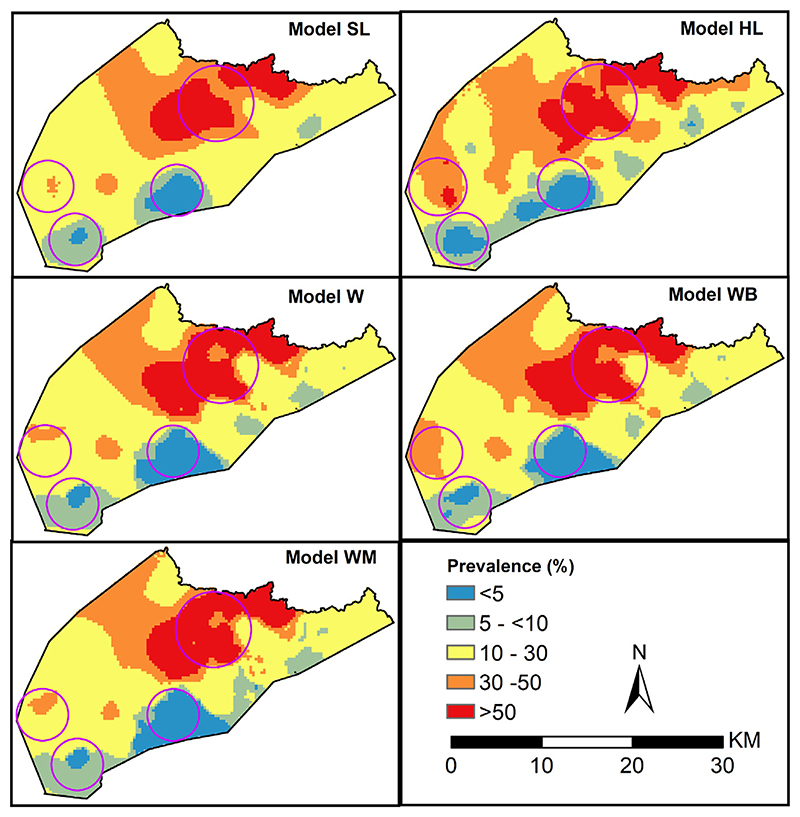
Predicted mean malaria prevalence at 0.3 × 0.3 km spatial resolution ranging from 0% (blue) to 83.2% (dark red) in 2010 for 5 geostatistical models based on school location (*SL*), gold standard model *(HL)* based on household location and three models accounting for locations uncertainty *W, WB, WM*. Circles show areas with differences.

All the five geostatistical models provide a similar spatial pattern of malaria prevalence in 2010 (Fig. 7). Overall, the malaria prevalence in the area is highly variable. The north-western region had the highest predicted prevalence of over 30% with a contiguous area of over 50%. The rest of the areas had prevalence of between 10% and 30% and a few areas in the southern and eastern border with less than 10% (Fig. 7). The gold standard model (*model SL*), had a maximum prevalence of 83.2%, the highest recorded among the five models in the area. This prevalence was closer to the maximum values recorded by the models accounting for uncertainty (75.3%, 77.4%, 76.0% for *models W, WB and WM*, respectively) relative to model (*SL*) estimate of 70.8%.

When we spatially overlaid population distribution maps with the predicated prevalence for each model, there were differences in the proportion of school-going children living in high malarious areas (at least 30% prevalence) in 2010. A third (33.1%) of the school-going children lived in areas with a prevalence of at least 30% based on model HL. The WB model had the closest proportion (27%) to model HL while, models SL W, and WM yields the lowest proportion of children within similar margins, 24.1%, 24.4% and 23.2%, respectively.

**Fig. 8:**
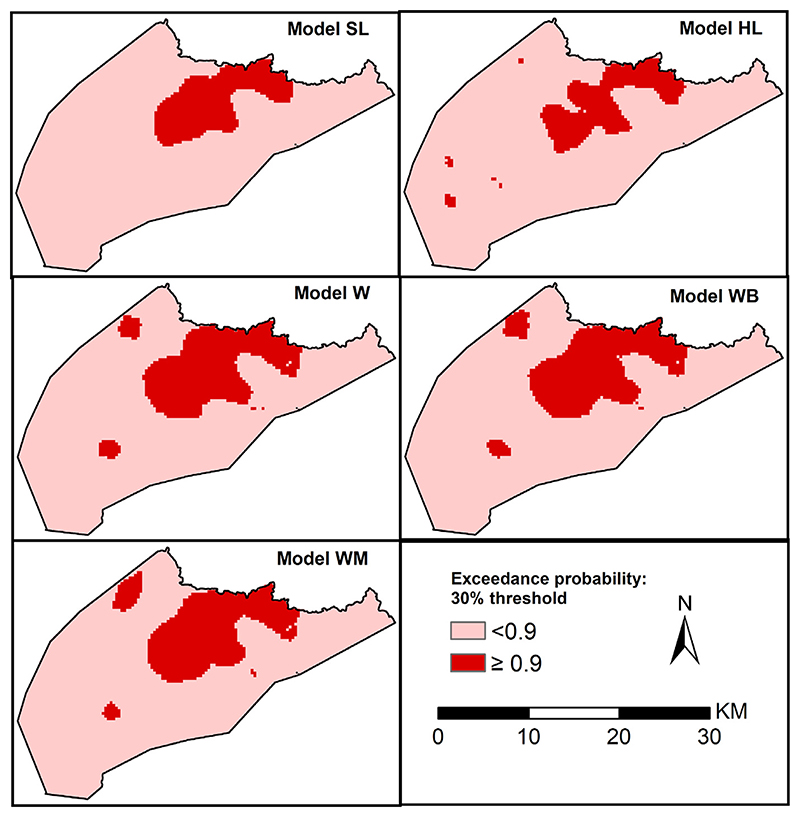
Maps of exceedance probability for a 30% prevalence threshold with a 90% probability on a 0.3 km regular grid for 5 geostatistical models based on school location (*SL*), gold standard model (*HL*) based on household location and three models accounting for locations uncertainty, models *W, WB and WM*.

Similarly, we also compared EPs that malaria prevalence lies above 30%. In Fig. 8, we highlight areas (in red) that are at least 90% likely to exceed 30% which were predominant in the north-west region. All the models identify a large contiguous area meeting the criterion in north-west region. However, the contiguous area has smaller geographical extents for model HL relative to the other 4 models. In addition to the large area, the models HL, W, WB and WM identify other smaller patches meeting the criteria which are not identified by the school location model. Approximately 10.3% of the school going children resided in areas where prevalence was over 30% with a probability of at least 90% based on models HL and SL. The models accounting for location uncertainty, W, WB and WB had slightly higher proportion, 16.7%, 16.8%, and 15.8% of the school going children, within the same margins, respectively.

Table 5 shows the average root-mean-square and bias metrics (see Section 4.2) which are used to compare how well each of the four considered models can yield a predictive risk surface for prevalence that more closely follows most the one generated by the geostatistical model based on the actual residence locations of the students. The results indicate that, albeit marginally, the model based on the school locations generates a predictive prevalence surface that better approximate the predictions generated by the HL model than the other three models that use school catchment areas. This can be explained in this case by the fact that the accuracy with which estimated catchment areas can approximate the actual catchment areas varies greatly across the study site. Figure S2.3 to S2.5 in *Supplementary material 2* shows an overlay of the modelled SCAs and the actual household location. Overall, the models fairly approximated the actual SCAs represented by household locations. Model W had the highest number of household within modelled SCAs (74.4%) while model WM had the least (68.8%) as shown in Table 1 of Supplementary material 2. Per modelled SCA, the number of households within modelled SCA was highly variable ranging from only 22.8% to 100% of the households. The performance was especially poor where there were several schools in close proximity.

## 5 Discussion

In this paper, we have introduced a geospatial framework for the geostatistical analysis of school malaria surveys data with incomplete spatial information on the residential addresses where disease exposure occurs. The solution that we have introduced in the paper can be summarized in three main steps. The first step requires the formulation of a suitable statistical model for the unobserved residence locations of the children attending school. This was achieved by generating school catchment areas based on factors that affect travel to primary schools and sampling possible residential locations within the catchments. In the second step, we used the proposed statistical model for the unobserved residence locations to generate samples of residence locations to average the likelihood function and carry out parameter estimation. The third step consisted of carrying out prediction for disease prevalence by plugging-in the parameter estimates obtained in the second step and generating predictive samples of prevalence at selected prediction locations. This framework provides a statistically rigorous approach to propagate the uncertainty arising from the missingness of residence locations, which in standard analyses of school malaria data is reduced to the location of the school (Macharia et al., 2018; Runge et al., 2020; Stensgaard et al., 2011).

One of the main benefits of the proposed modelling framework is it provides a solution to several other statistical problems that have been covered in this paper: combining data from multiple surveys, some of which may have missing information on the residence of sampled individuals; reducing the bias in the estimation of regression relationships between prevalence and disease risk factors, induced by the aggregation of spatial information to a single location. However, as suggested by the results of the applications, the extent to which those issue can be successfully tackled is largely dependent on how well the school-catchment-area models allow for reliable inferences on the residence locations.

In our first case study, whilst areal-level summaries, such as the total population falling in areas with an exceedance probability over 90% for a 30% prevalence thresholds, were moderately similar (ranging between 23% and 14%) across the models considered, we found substantial localized differences in both the predicted prevalence (Fig. 5) and the exceedance probabilities (Fig. 6) between models that accounted for location-uncertainty *(models W, WB and WM*) and those that did not, based on school locations (*models SL and SLCA*. However small these areas may be, this aspect is especially important to consider when targeting and prioritizing specific areas with suitable malaria control activities. Among the models that accounted for location-uncertainty, the differences were less strong, suggesting that the assumptions made by different geographical access models may not strongly affect the inferences on disease prevalence.

In the second application, we assessed how similar are the inference between geostatistical models that incorporate school-catchment area models and geostatistical models that use the actual residence locations. The results suggested that inaccurate school catchment areas can have a material impact on geostatistical inferences and a simpler model that only uses the school locations can deliver better predictive performances. Future research should thus focus on improving the catchment area models which provide a way of developing more realistic geostatistical models than those simply use school locations. For example, in the first application, if more accurate information were available, such as the *ward* (the smallest administrative unit in Kenya) or enumeration area in which the students resided, the boundaries of these administrative areas could be used as an alternative to the modelled school catchment areas. However, the improvement accrued by exploiting this information will vary according to the size of those administrative areas relative to the school catchment area.

In both applications, among the three models considered for the school catchment area, the one assuming “walking only” (i.e. Model W) as a mode of travel to school is the one more strongly supported by previous studies. It is in fact more plausible that majority of the children walk to reach their school, since this mobility pattern has been observed in relation to other service points (e.g., healthcare providers) in Western Kenya (Salon and Gulyani, 2010; Dixit et al., 2016), as a result of low levels of ownership of motor vehicles (Macharia et al., 2021a). However, more recently, it has also been observed that the number of motorcycles, locally known also known as “boda boda”, have been increasing especially in Western Kenya (Macharia et al., 2021a), with a smaller proportion of children more likely to use the public transport or private vehicles. For these reasons, more accurate information on the mode of travel of children could help to significantly improve the methods presented in this paper, which can be applied to other infectious diseases that are monitored using school data, as in the case of helminth and schistosomiasis infections (Hodges et al., 2011; Tchuem Tchuenté et al., 2012; Soares Magalhães et al., 2011; Gitonga et al., 2010; Brooker et al., 2009; Ashton et al., 2015; Mathanga et al., 2015).

The framework developed is not only applicable to school survey data but also to other data-scenarios where there is missing information on the location where most of the exposure to the disease is likely to occur. For example, in some households surveys and routinely collected data, due to confidentiality reasons, the residence location cannot be made available for analysis. An example of this is given by the Demographic and Health Survey (DHS). DHS are nationally representative household surveys that have been conducted in more than 85 countries since 1984 to collect demographic and health data (Corsi et al., 2012). However, to reduce disclosure risk in DHS, a cluster is assigned the coordinates of the centre of the sampled enumeration area and further randomly displaced. The framework developed can be adapted to partially account for this geomasking. This is vital because in low resource settings, such household surveys are the only source of development indicators. An important aspect of the proposed framework is that it can also be used to combine data from multiple sources with varying accuracy for the information of the residence of the sampled individuals.

As shown in this study, catchment areas models are an integral component of disease mapping and are essential in order to yield reliable inferences and summaries of uncertainty for the health outcome under investigation (Macharia et al., 2021b). While the three types of catchment areas we generated did not yield substantially different results, further research is required to improve these models. Here, each of the models assumed non overlapping catchments but, in reality, students from the same location can attend different schools and thus create overlapping catchments. To accommodate this, the attractiveness factor should not only be a function of distance or travel time, as in our case, but should account for school capacity, classroom size, number of teachers, perceived teaching quality, and previous examinations results. In our analysis, every student was assumed to attend their nearest school. This could be further improved if school attendance data were available, which would allow us to empirically identify a threshold for the distance or travel time below which students do in fact go to the nearest school. Such an approach has been implemented in the construction of health facility catchment areas by using health seeking behaviour information from DHS (Alegana et al., 2012).

In addition to the limitations outlined in the previous section, there are other limitations in the illustrated applications. In our analysis, we excluded boarding schools, which may lead to the underestimation of the travel time and thus overestimate the size of SCAs. However, there were only nine schools that were purely boarding out of 2170 in Western Kenya in 2009. The access models were parametrized using speeds and modes of transport from other studies (Samimi and Ermagun, 2013; Macharia et al., 2017; Mehdizadeh et al., 2017; Alegana et al., 2021) since we did not have data for Western Kenya. Future studies should consider collecting data on mode of transport, speeds and the utilized school in this region for improved modelling of accessing metrics and SCAs. The chosen travel route to school is a complex process influenced by socio-economic factors and facilitators of movement such as roads and obstacles. We assumed that students do not bypass their nearest school, but a small proportion likely bypass their nearest school due to poverty, affordability, or parents’ past education experience among other factors.

## Supplementary Material

Supplementary material 1

Supplementary material 2

## Figures and Tables

**Table 1:** Summary of the covariates in the analysis of Section 3.

Covariate	Description	Source and resolution
1. Annual mean Temperature	Temperature affects the survival and development of *P. falciparum* from larvae into viable adults. Temperatures greater than 34 °C lead to almost 100% larval mortality, while temperatures less than 16 °C, the larvae are unable to produce viable adults (Bayoh and Lindsay, 2004). Mortality of the anopheles mosquitoes increases at ambient temperatures approaching 40 °C while temperatures between 25 °C and 30 °C are considered optimum for *Plasimodium falciparum* sporogony (Molineaux, 1988).	MODIS, 5.6 km grids (Busetto and Ranghetti, 2016)
2. Annual mean Precipitation	Rainfall combined with suitable ambient temperatures provides potential breeding environments for Anopheles (Noor et al., 2014; Dutta and Dutt, 1978).	CHIRPS, 5 km grids (Funk et al., 2015).
3. Urbanization	Malaria infection is usually lower in urban compared to rural areas due to reduced malaria vector density and biting rate (Kabaria et al., 2017). Urbanization was proxied by nighttime lights (NTL) (Savory et al., 2017). NTL are also associated with human activity, population distribution, reduced poverty rates and increased access to health facilities (Kabaria et al., 2017).	NTL 1 km square grids (Savory et al., 2017)
4. Enhanced vegetation index	Vegetation acts as a proxy for the presence of suitable mosquitoes breeding sites (Noor et al., 2014; Dutta and Dutt, 1978).	MODIS, 0.25 km grids (Busetto and Ranghetti, 2016)

**Table 2:** Summary of factors that affect travel time to schools in Western Kenya.

Factor	Description	Type, Year and Resolution
1. Road Network	The road network was based on data from the Ministry of Transport that used the gold GPS technique to map coverage of roads in 2016. The layer was updated via OpenStreetMap and Google Map Maker and was cleaned by deleting duplicates and correcting digitization errors such as overshoots and undershoots at connection points or junctions and those that extended into water bodies. Further details in Macharia et al. (2017, 2021a) and Joseph et al. (2020).	Vector layer, 2016
2. Land use	The land use/cover information was obtained from 2016 Copernicus Sentinel-2 satellite containing five classes (bare areas,built-up areas, water bodies, cultivated and vegetation cover areas available at http://geoportal.rcmrd.org/	Raster, 20 by 20 m. 2016.
3. Digital elevation model (DEM)	The slope of the terrain was derived from a DEM based on Shuttle Radar Topographic Mission (SRTM) available at http://geoportal.rcmrd.org/	Raster, 30 by 30 m
4. Barriers to movement	Barriers to movement included major rivers, lakes, flooded areas and protected areas (Joseph et al., 2020).	Vector layers.

**Table 3:** Speeds assigned to generate catchments for Models W (Walk only), WB (Walk and Bicycle) and WM (Walk and motorized). All speeds are in kilometer/hour and take a walking mode of transport unless where otherwise stated.

Land and road type	Model W	Model WB	Model WM
Tree cover	3.5	3.5	3.5
Shrub cover	4.5	4.5	4.5
Grassland	4	4	4
Cropland	3.5	3.5	3.5
Regularly flooded	0	0	0
Sparse vegetation	4.5	4.5	4.5
Bare areas	5	10 (*Bicycling*)	5
Built up areas	5	10 (*Bicycling*)	5
Open water	0	0	0
Primary road	5	5	50 (*Motorized*)
Secondary road	5	5	30 (*Motorized*)
County road	5	10 (*Bicycling*)	25 (*Motorized*)
Rural road	5	10 (*Bicycling*)	5

**Table 4:** Summaries of the simulation study of the application presented in Section 3; for more details see Section 3.3.

Model	Bias	RMSE	Sensitivity	Specificity
W	0.002	0.176	0.741	0.757
WB	−0.005	0.190	0.716	0.729
WM	−0.008	0.257	0.569	0.581
SL	−0.001	0.169	0.764	0.756
SLCA	−0.001	0.169	0.765	0.756

**Table 5:** Average root-mean-square-error (RMSE) and bias as specified in Section 4.2.

Model	Average RMSE	Average bias
SL	0.071	0.005
W	0.082	0.007
WB	0.085	0.007
WM	0.082	0.007

## Data Availability

Data that support the findings of this study are available at Population Health Dataverse, http://dx.doi.org/10.7910/DVN/UQLTO5, generated gridded surfaces are located at https://doi.org/10.6084/m9.figshare.19804207.v1. The codes used in this analysis are available at https://github.com/giorgilancs/mbgmissinglocations.
