# Supplementary material for: Combining school-catchment area models with geostatistical models for analysing school survey data from low-resource settings: Inferential benefits and limitations
Source: Spat Stat. Author manuscript; Available in PMC 2022 Oct 1. (PMC7613137; doi:10.1016/j.spasta.2022.100679)
Supplement: Supplementary material 1 [file EMS147098-supplement-Supplementary_material_1.pdf]

# **Supplementary material 1**: Combining school-catchment area models with geostatistical models for analysing school survey data from low-resource settings: inferential benefits and limitations

Peter M. Macharia[a,b,*], Nicolas Ray[c,d], Caroline W. Gitonga[b], Robert W. Snow[b,e], Emanuele Giorgi[a]

[a]*Centre for Health Informatics, Computing, and Statistics, Lancaster Medical School, Lancaster University, Lancaster, LA1 4YW, UK*
[b]*Population Health Unit, Kenya Medical Research Institute-Wellcome Trust Research Programme, PO, Box 43640, Nairobi, Kenya*
[c]*GeoHealth group, Institute of Global Health, University of Geneva, Geneva, Switzerland*
[d]*Institute for Environmental Sciences, University of Geneva, Geneva, Switzerland*
[e]*Centre for Tropical Medicine and Global Health, Nuffield Department of Medicine, University of Oxford, Oxford, OX3 7LG, UK*

## 1. Datasets

The spatial layers used to compute geographic access and school catchment areas; schools, road network, land use and digital elevation model. The schools database consisted of a geocoded listing of schools in Kenya developed through a nationwide mapping survey in 2009 Mulaku and Nyadimo (2011). There were, 15,439 schools in western Kenya with circa 4,000 being public primary schools (PPS). After removing duplicates and erroneous entries, 2,137 schools were within the study region. After including, other PPS in the region that were part of the 2009 malaria survey Gitonga et al. (2010), a total of 2170 schools were included in the study (Figure S1.1).

*Corresponding author
*Email address:* `pmacharia@kemri-wellcome.org` (Peter M. Macharia)

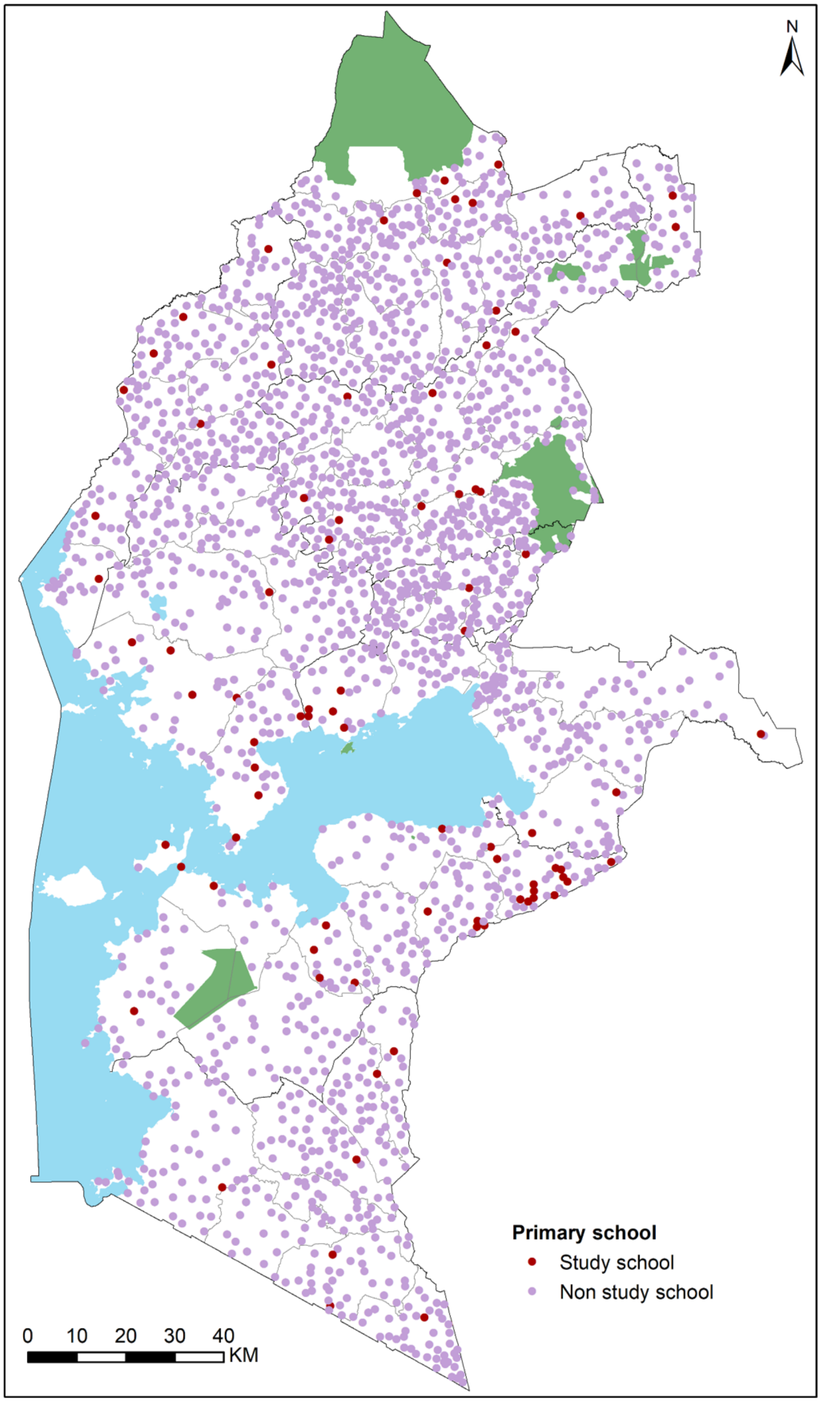


Figure S1.1: All- 2170- primary public day schools in the study area and with those included in the study colored red.

To account for on road movement for the students (walking, cycling, and motorized transport) we leveraged on previously assembled databases of road for this region Macharia et al. (2021); Joseph et al. (2020); Macharia et al. (2017) (Figure S1.2).

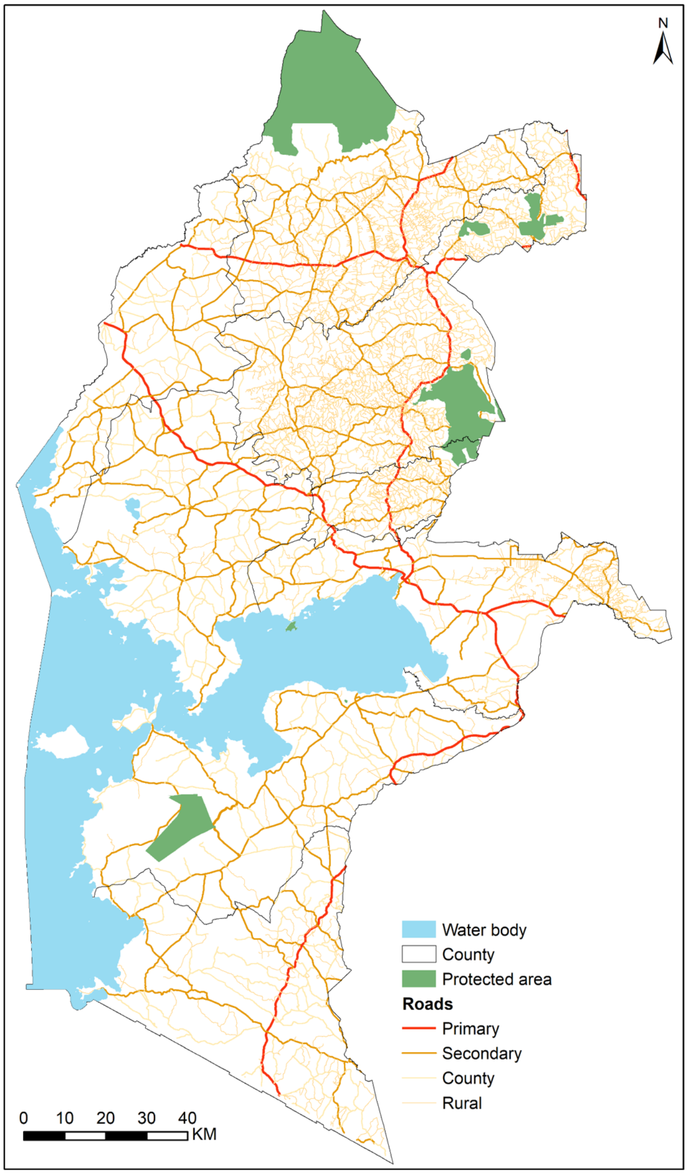


Figure S1.2: Road network in western Kenya.

Land cover layer to represent the travel impedance in spaces between the roads was obtained from RCMRD Geoportal (http://geoportal.rcmrd.org/) at 20 metres spatial resolution based on the 2016 Copernicus Sentinel-2 satellite(Figure S1.3).

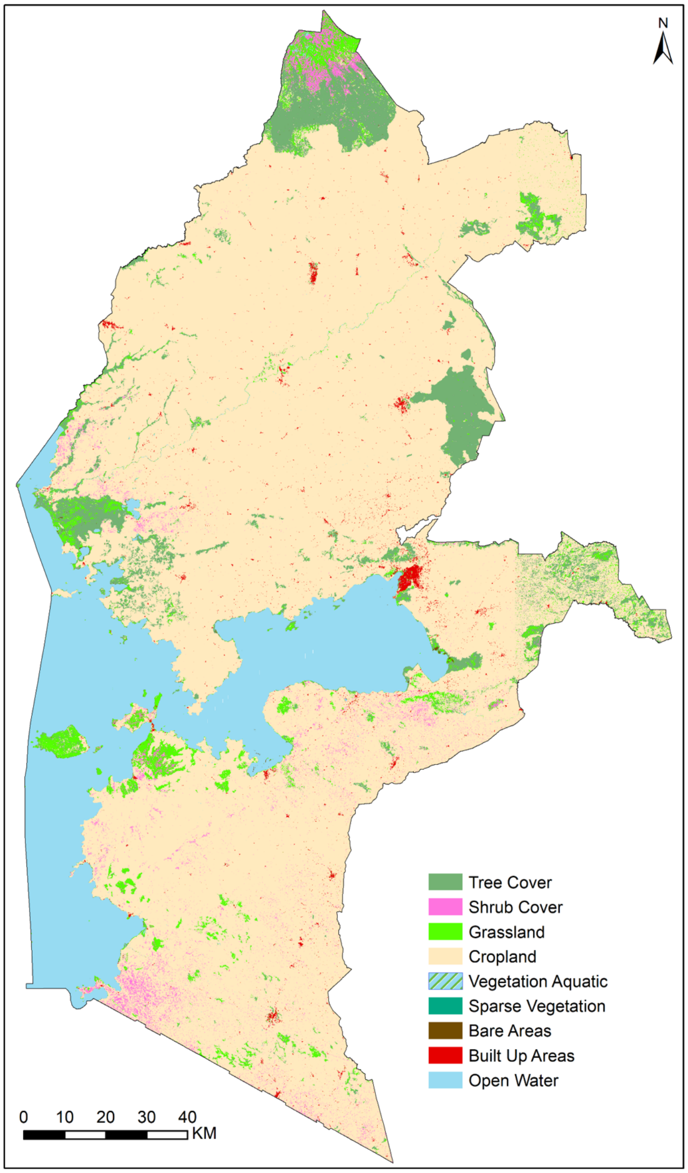


Figure S1.3: Land use/cover in western Kenya.

A digital elevation (DEM) was used to derive slope that impedes walking and bicycling speeds. It was as obtained from RCMRD Geoportal (http://geoportal.rcmrd.org/) at 30 metres spatial resolution based on based on Shuttle Radar Topographic Mission (SRTM)(Figure S1.4).

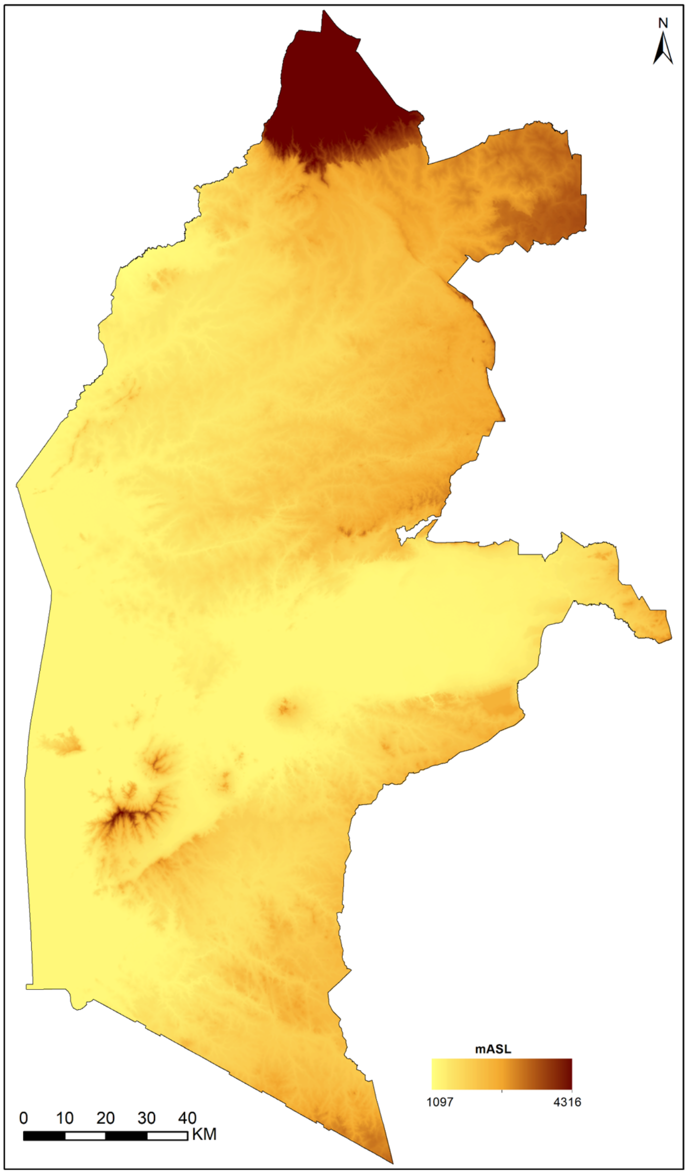


Figure S1.4: Elevation variation in western Kenya.

## 2. Geographic Access

Spatial accessibility based on three travel models showing travel time to the nearest school, Figure S1.5 is the walking model ($W$), Figure S1.6 is the combination of walking and bicycling model ($WB$) while Figure S1.7 is the combination of walking and motorised transport($WM$)

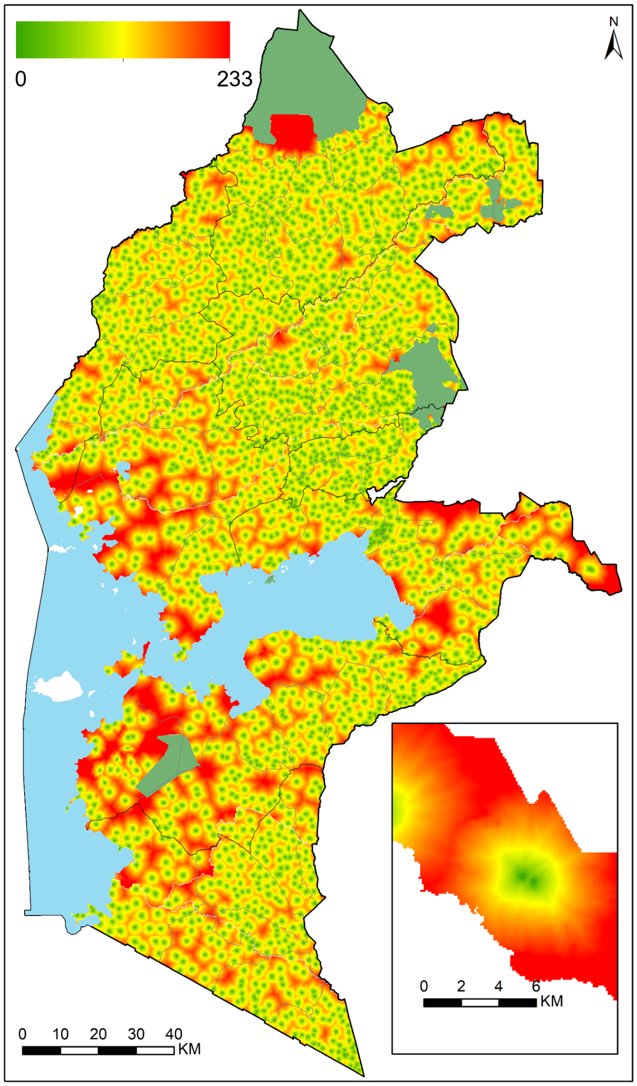


Figure S1.5: Travel time to the nearest school based on walking colored coded from green (0 minutes) to red (233 minutes) in western Kenya.

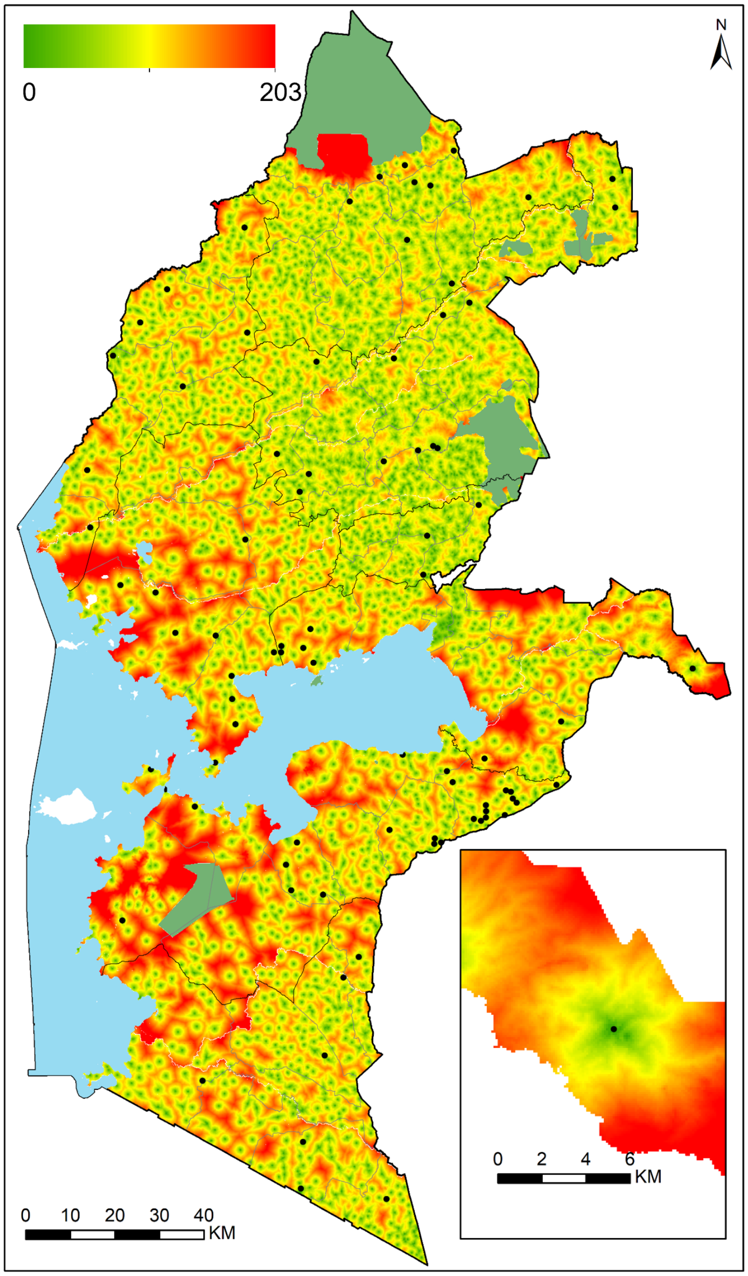


Figure S1.6: Travel time to the nearest school based on walking/bicycling model colored coded from green (0 minutes) to red(203 minutes) in western Kenya.

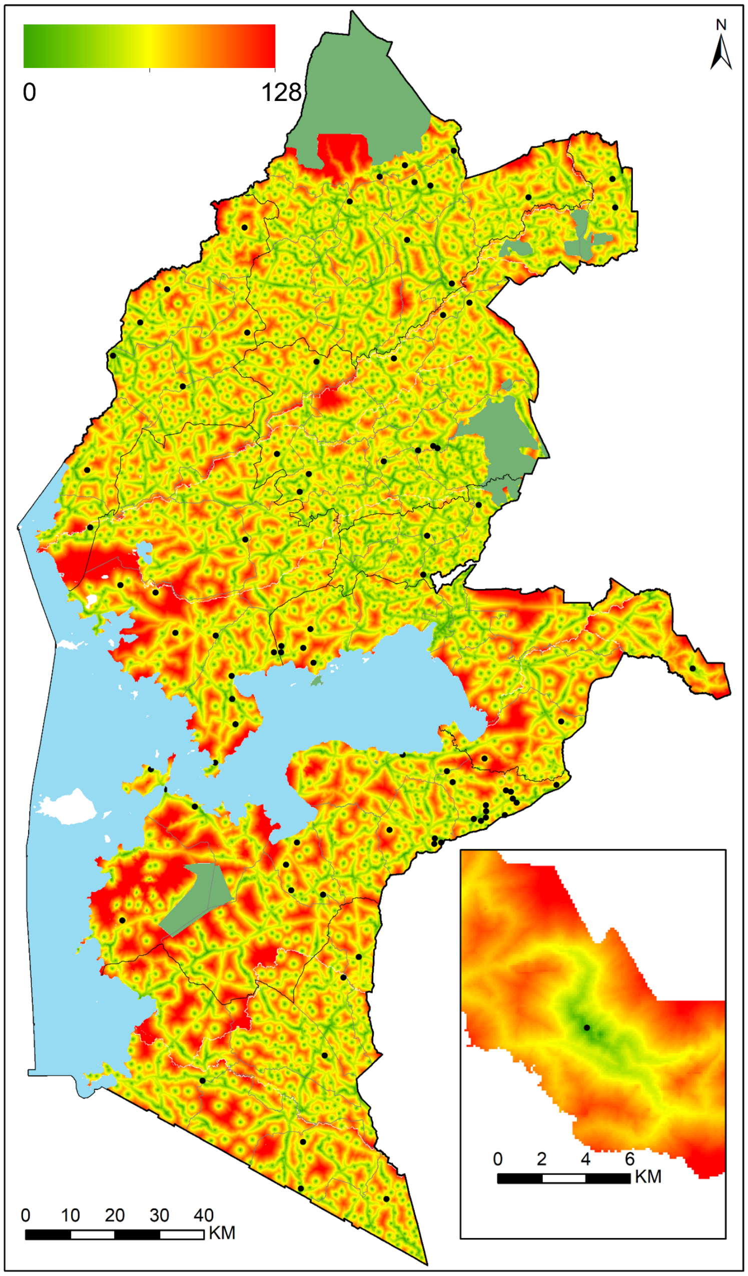


Figure S1.7: Travel time to the nearest school based on walking/motorized model colored coded from green (0 minutes) to red(123 minutes) in western Kenya.

### School catchment area

School catchment areas were modelled for all 2170 schools and subset for 84 sampled primary schools in western Kenya.

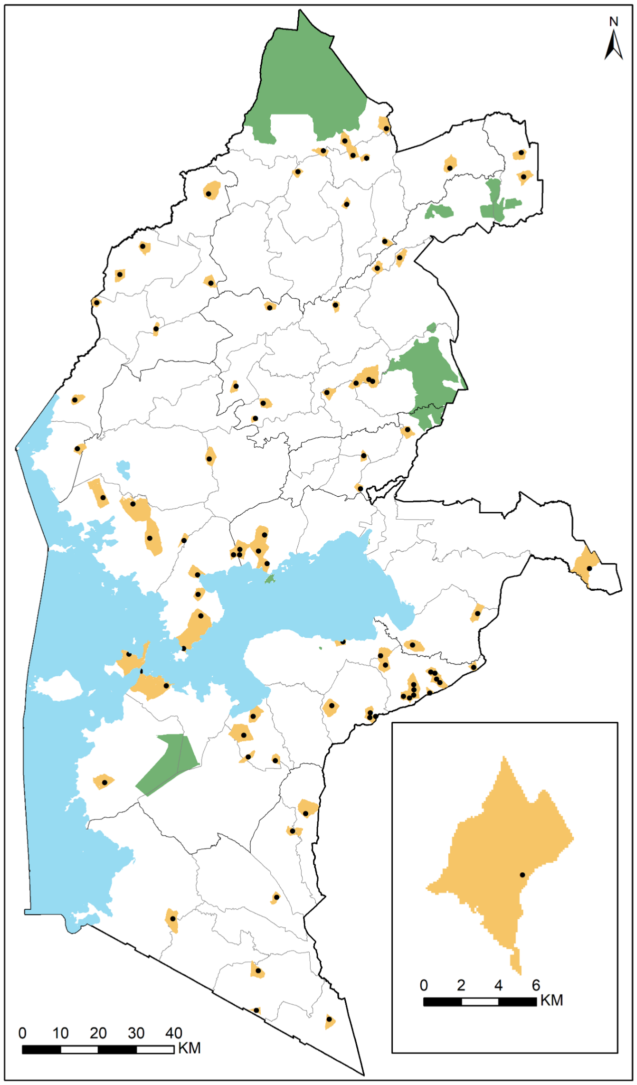


Figure S1.8: Eighty-four school catchment areas based on walking model of transport ($W$) in western Kenya for the sampled schools.

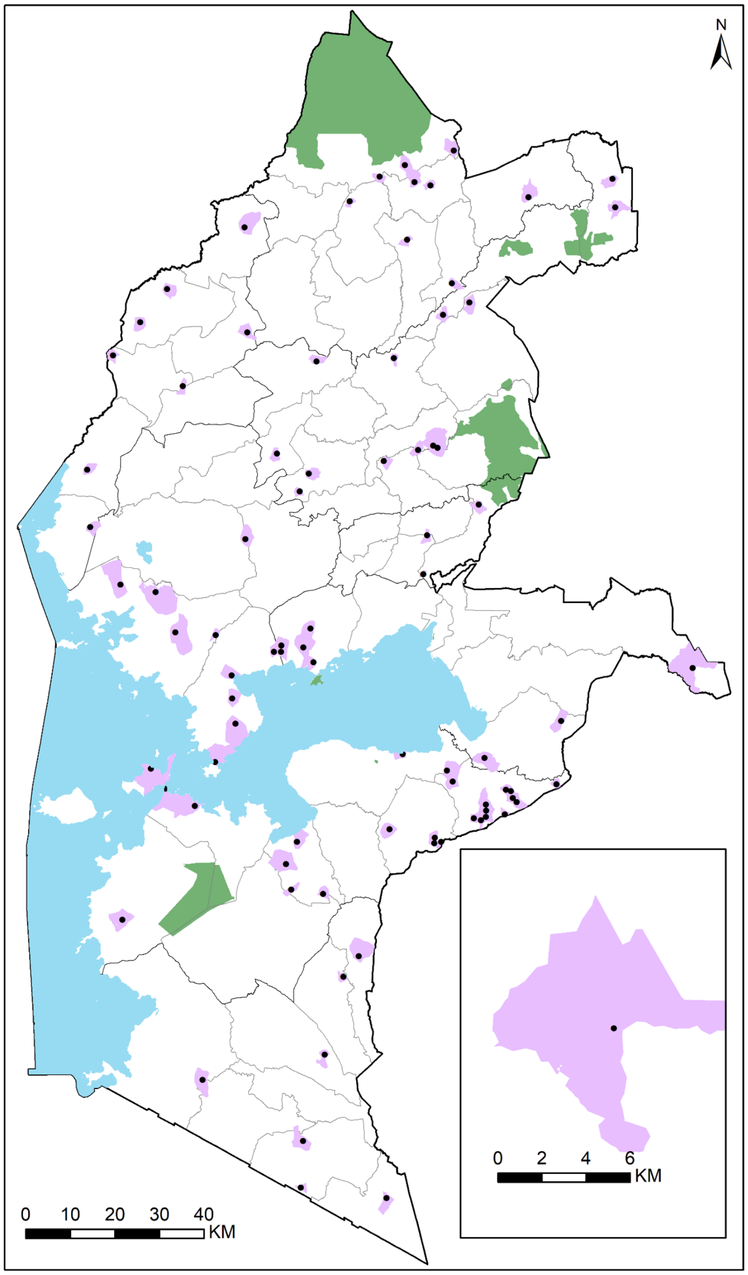


Figure S1.9: Eighty-four school catchment areas based on walking and bicycling model of transport($WB$) in western Kenya for the sampled schools.

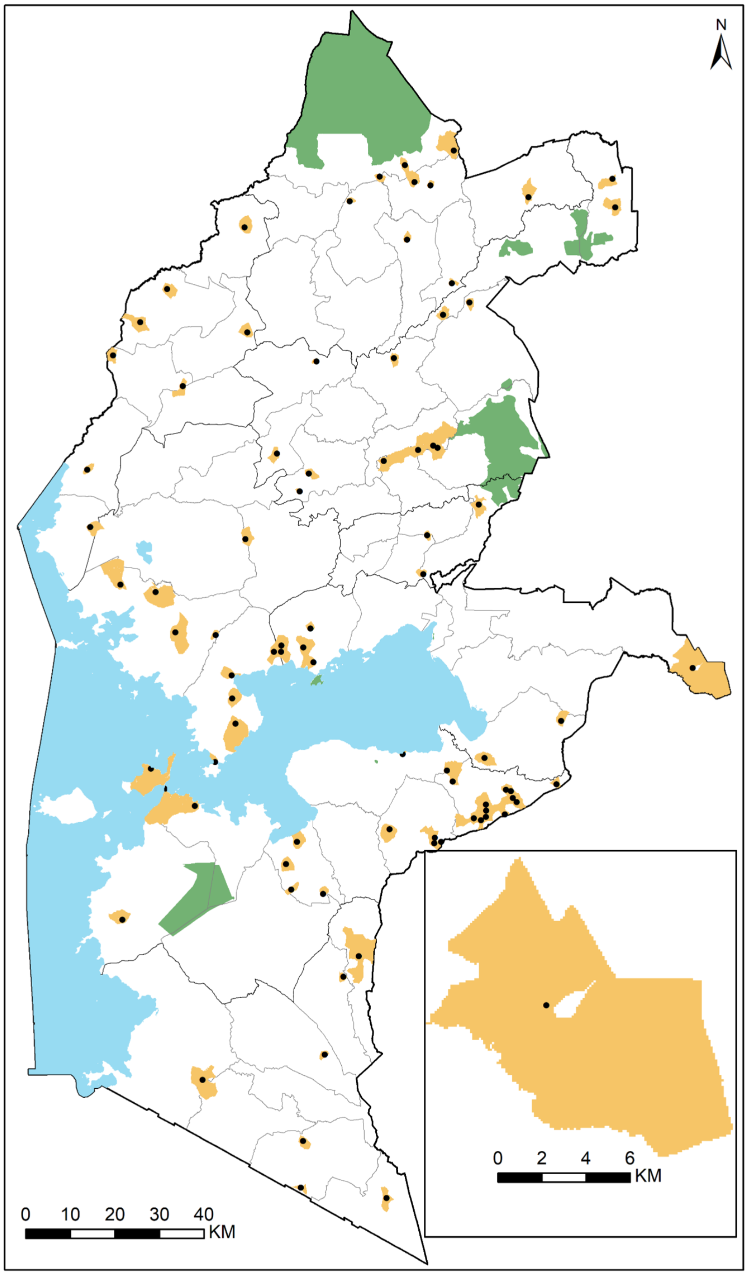


Figure S1.10: Eighty-four school catchment areas based on walking and motorized model of transport ($WM$) in western Kenya for the sampled schools.

## Explanatory analysis

Enhanced vegetation index(EVI) was excluded from the analysis, since this found to be highly correlated (Figure S1.11) with both the precipitation and the temperature. The three remaining covariates, temperature, precipitation and night time lights(NTL) (Figure S1.11) showed an approximately linear relationship with the empirical logit and were were used as predictors the geostatistical models.

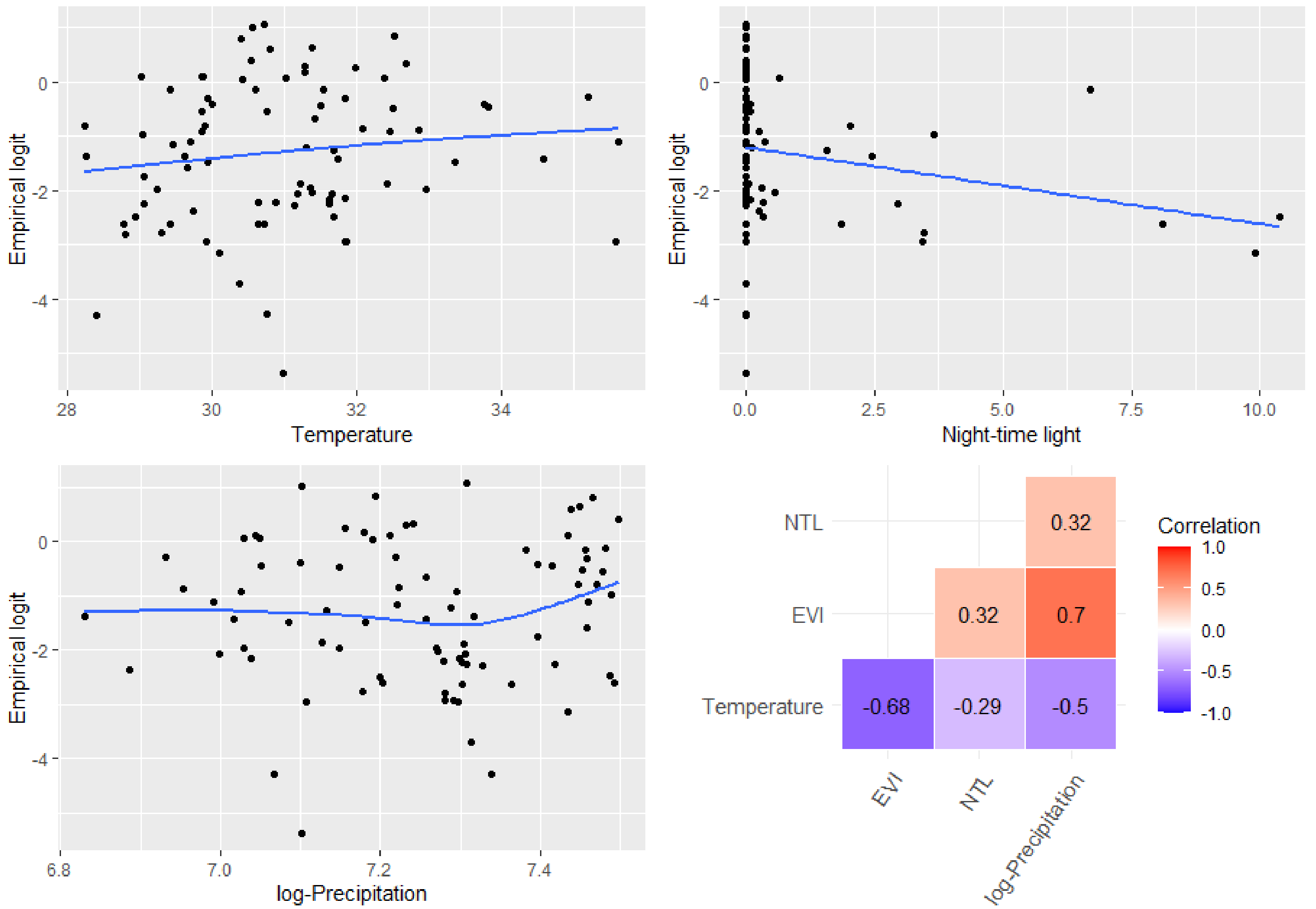


Figure S1.11: Scatter plots of the empirical logit-transformed prevalence against temperature, night-time light (NTL), log-precipitation. The solid blue lines are natural linear splines. The lower right panel shows the empirical correlation between each pair of covariates supporting whu EVI was exluded.

## Mesh

Figure S1.12 shows the mesh used to define the piece-wise linear approximation of the Gaussian field $S(x)$

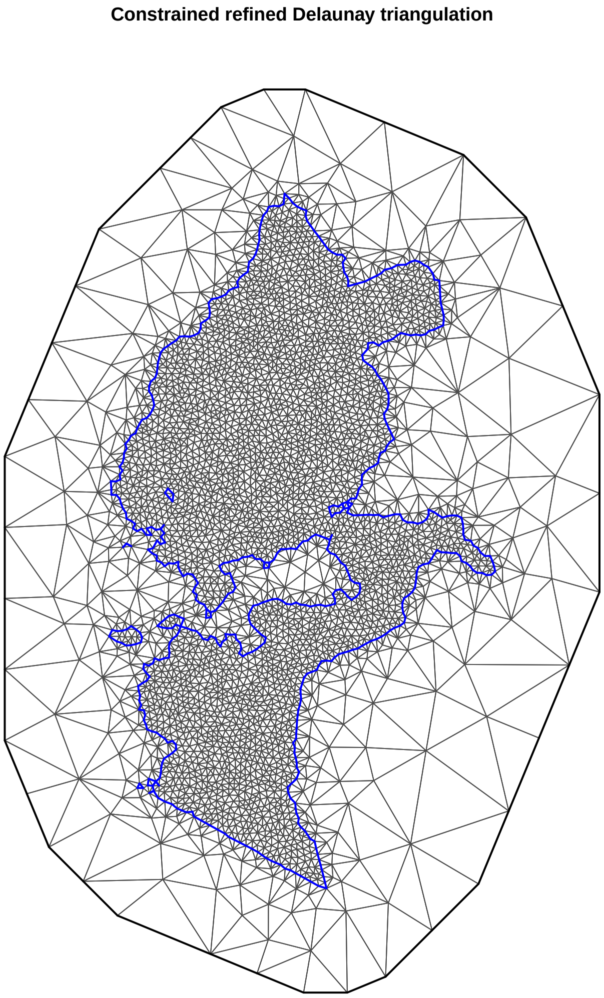


Figure S1.12: Mesh generated using the `inla.mesh.2d` function from the `INLA` R package (Krainski et al., 2018).

## References


Gitonga, C.W., Karanja, P.N., Kihara, J., Mwanje, M., Juma, E., Snow, R.W., Noor, A.M., Brooker, S., 2010. Implementing school malaria surveys in Kenya: Towards a national surveillance system. Malaria Journal 9, 1–13. doi:10.1186/1475-2875-9-306.

Joseph, N.K., Macharia, P.M., Ouma, P.O., Mumo, J., Jalang'o, R., Wagacha, P.W., Achieng, V.O., Ndung'u, E., Okoth, P., Muñiz, M., Guigoz, Y., Panciera, R., Ray, N., Okiro, E.A., 2020. Spatial access inequities and childhood immunisation uptake in Kenya. BMC public health 20, 1407. doi:10.1186/s12889-020-09486-8.

Krainski, E., Gómez Rubio, V., Bakka, H., Lenzi, A., Castro-Camilo, D., Simpson, D., Lindgren, F., Rue, H., 2018. Advanced Spatial Modeling with Stochastic Partial Differential Equations Using R and INLA. doi:10.1201/9780429031892.

Macharia, P.M., Mumo, E., Okiro, E.A., 2021. Modelling geographical accessibility to urban centres in Kenya in 2019. Plos One 16, e0251624. doi:10.1371/journal.pone.0251624.

Macharia, P.M., Odera, P.A., Snow, R.W., Noor, A.M., 2017. Spatial models for the rational allocation of routinely distributed bed nets to public health facilities in Western Kenya. Malaria Journal 16, 367. doi:10.1186/s12936-017-2009-3.

Mulaku, G., Nyadimo, E., 2011. Gis in education planning: The kenyan school mapping project. Survey Review 43, 567–578.
