# Supplementary material for: Combining school-catchment area models with geostatistical models for analysing school survey data from low-resource settings: Inferential benefits and limitations
Source: Spat Stat. Author manuscript; Available in PMC 2022 Oct 1. (PMC7613137; doi:10.1016/j.spasta.2022.100679)
Supplement: Supplementary material 2 [file EMS147098-supplement-Supplementary_material_2.pdf]

Supplementary material 2: Combining school-catchment area models with geostatistical models for analysing school survey data from low-resource settings: inferential benefits and limitations

Peter M. Macharia[a,b,*], Nicolas Ray[c,d], Caroline W. Gitonga[b], Robert W. Snow[b,e], Emanuele Giorgi[a]

[a]*Centre for Health Informatics, Computing, and Statistics, Lancaster Medical School, Lancaster University, Lancaster, LA1 4YW, UK*
[b]*Population Health Unit, Kenya Medical Research Institute-Wellcome Trust Research Programme, PO, Box 43640, Nairobi, Kenya*
[c]*GeoHealth group, Institute of Global Health, University of Geneva, Geneva, Switzerland*
[d]*Institute for Environmental Sciences, University of Geneva, Geneva, Switzerland*
[e]*Centre for Tropical Medicine and Global Health, Nuffield Department of Medicine, University of Oxford, Oxford, OX3 7LG, UK*

*Corresponding author
Email address: `pmacharia@kemri-wellcome.org` (Peter M. Macharia)

Preprint submitted to Spatial Statistics *March 28, 2022*

1. Datasets

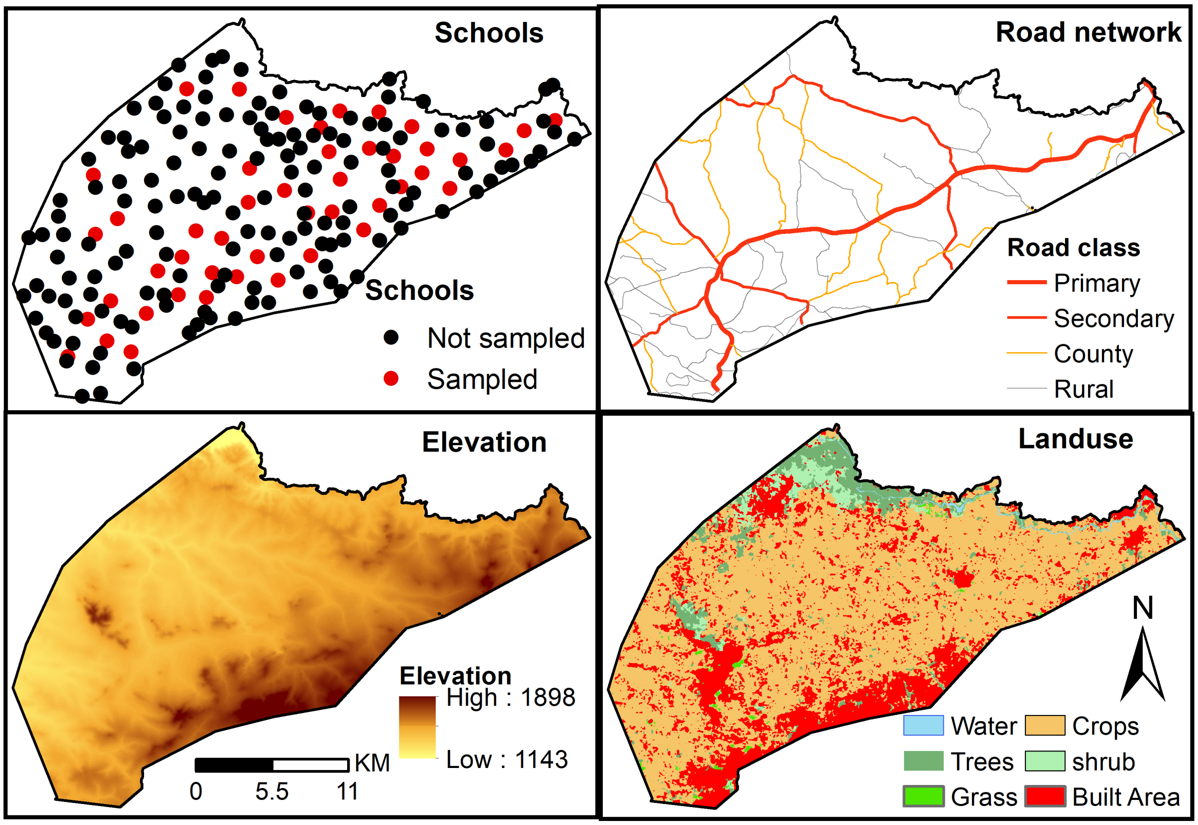

Figure S2.1: The spatial layers used to compute geographic access and school catchment areas: school locations and factors that affect travel

2. Travel time and school Catchment areas (SCAs)

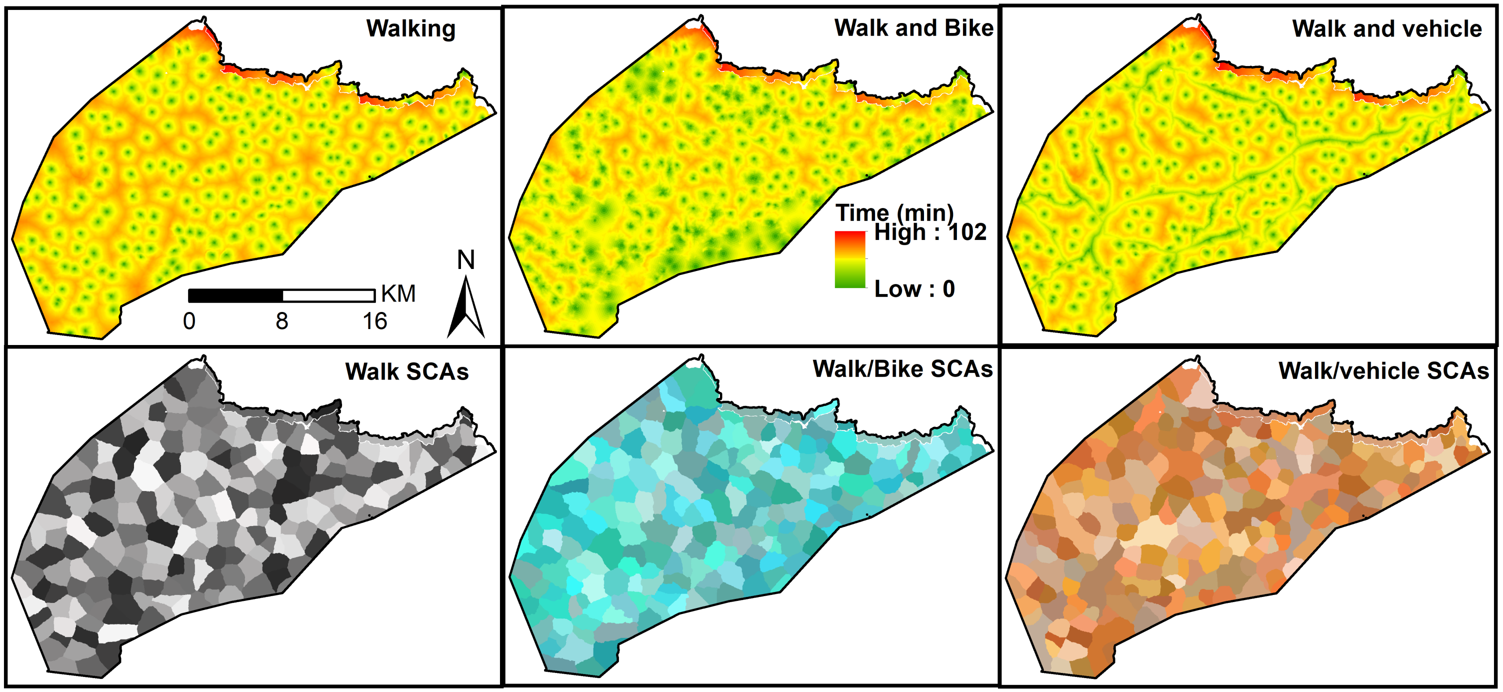

Figure S2.2: Travel time (in minutes) to the nearest primary school based on three travel scenarios (W, WB and WM) and and their corresponding SCAs

SCA and household locations

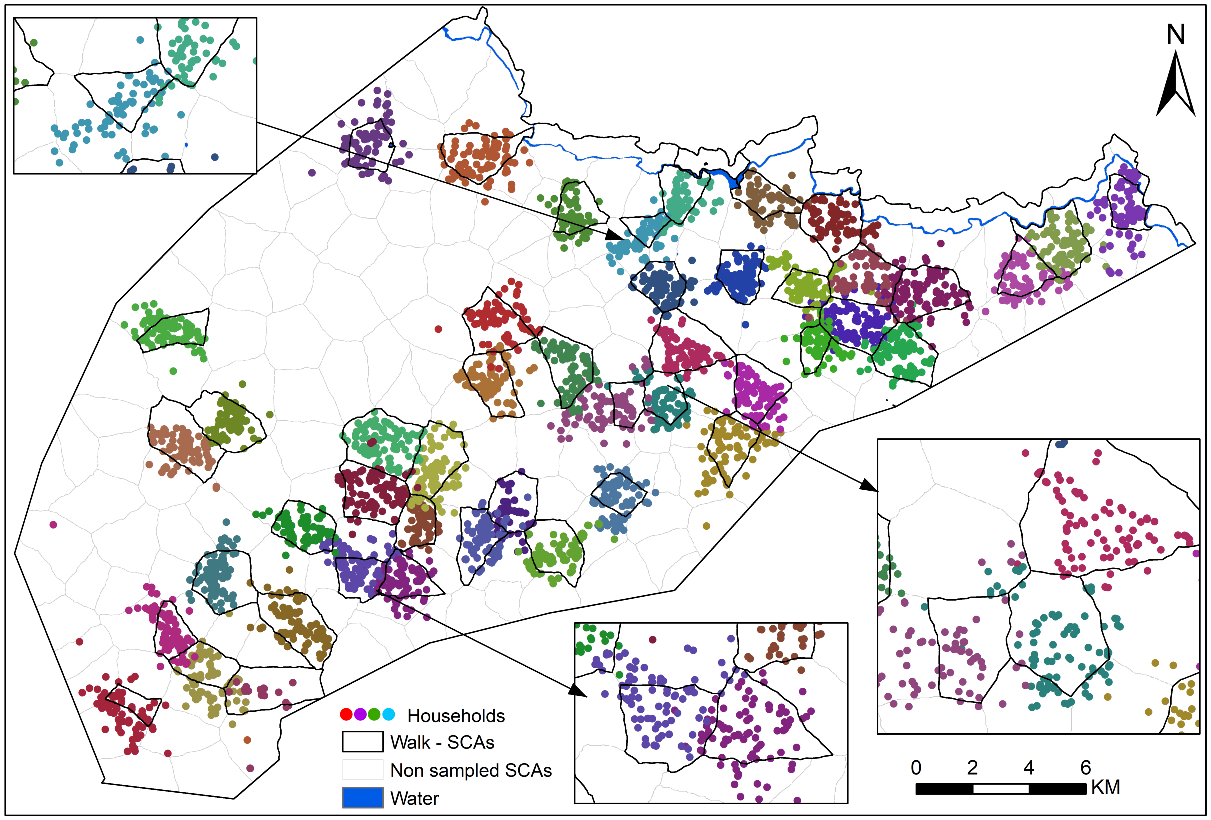

Figure S2.3: Spatial overlay of modelled SCAs (model W- walking only scenario) and household location of school-going children.

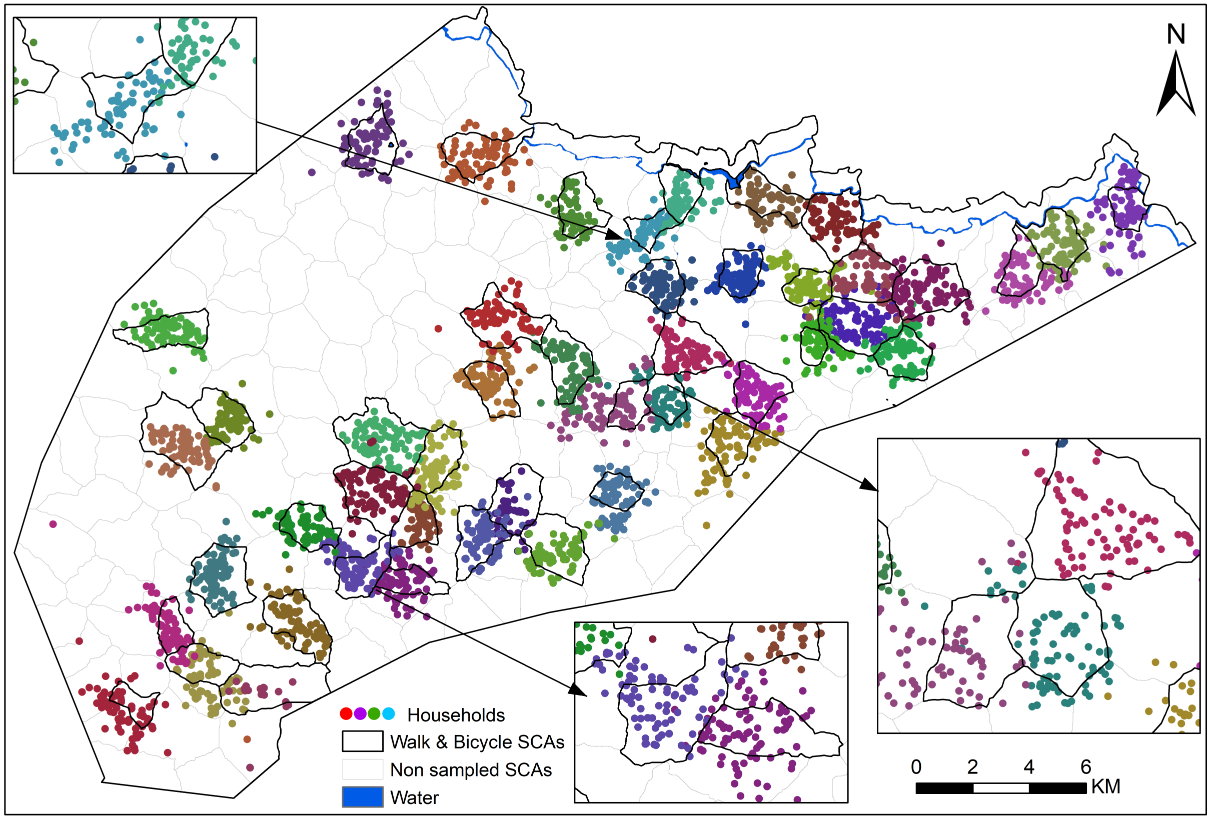

Figure S2.4: Spatial overlay of modelled SCAs (model WB- walking/bicycling scenario) and household location of school-going children.

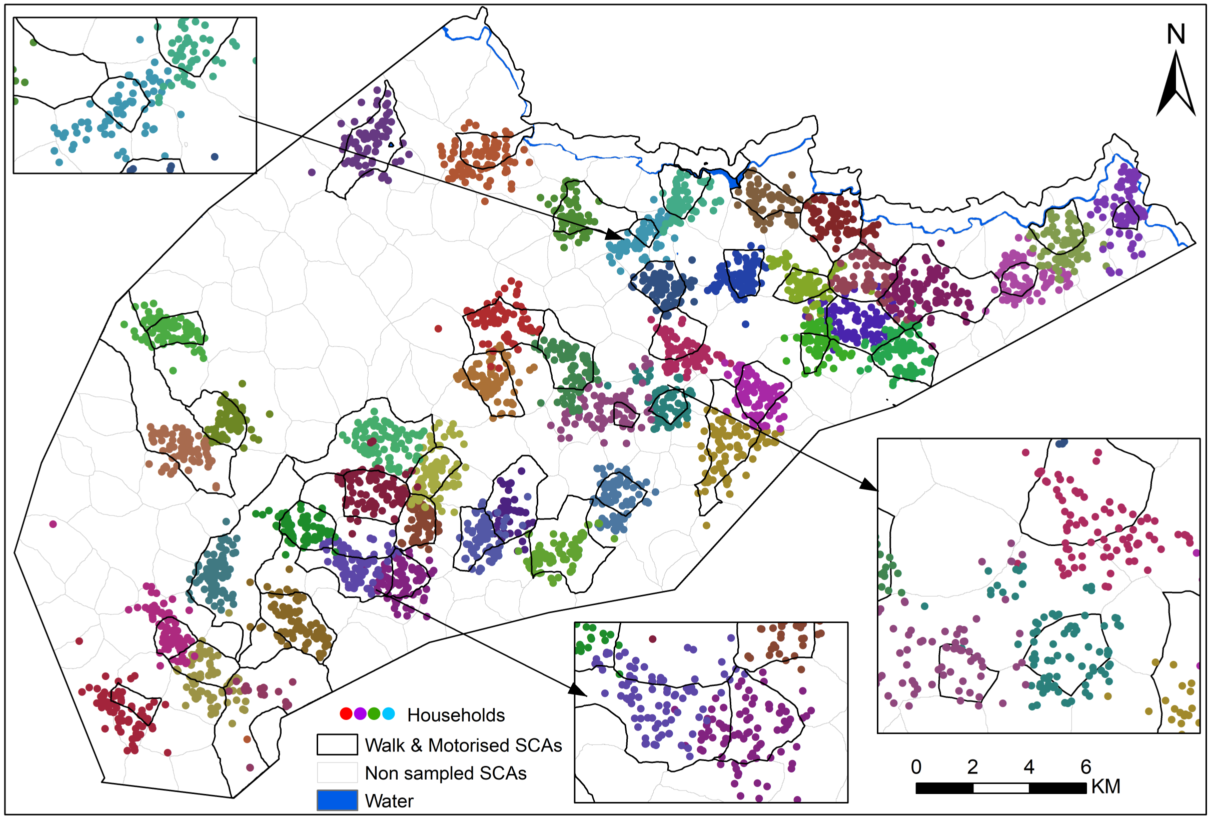

Figure S2.5: Spatial overlay of modelled SCAs (model WM-walking/motorized scenario) and household location of school-going children.

Table S2.1: Proportion of children within modelled SCAs from the overlay SCAs and geolocated household locations for Model W (Figure S2.3), Model WB (Figure S2.4) and Model WM (Figure S2.5)

Model	HHs within SCAs (%)	Range of HHs within SCAs per school (%)	% of SCAs with ≥50% HHs	% of SCAs with ≥70% HHs	% of SCAs with ≥90% HHs
W	74.4	27.7%– 99.1 %	89.1%	67.4%	19.6%
WB	72.8	29.8% – 99.1%	82.1%	63.0%	17.4%
WM	68.8	22.8% - 100%	76.1%	56.5%	21.7%

Mesh

Figure S2.6 shows the mesh used to define the piece-wise linear approximation of the Gaussian field $S(x)$

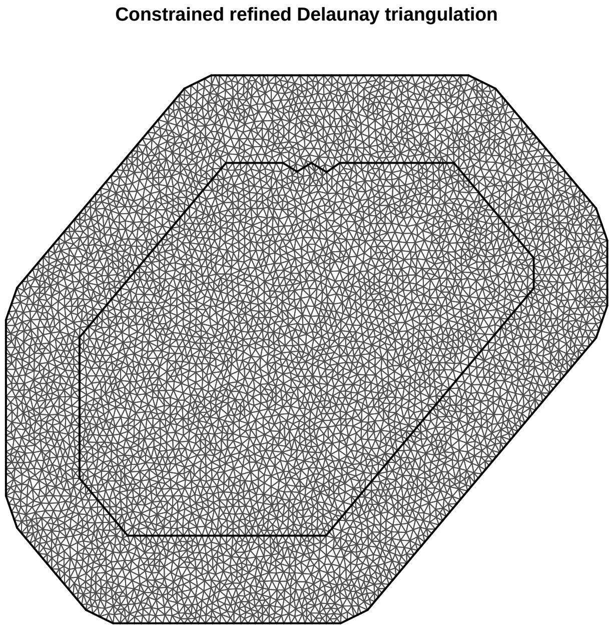

Figure S2.6: Mesh generated using the `inla.mesh.2d` function from the `INLA` R package (Krainski et al., 2018).

References

Krainski, E., Gómez Rubio, V., Bakka, H., Lenzi, A., Castro-Camilo, D., Simpson, D., Lindgren, F., Rue, H., 2018. Advanced Spatial Modeling with Stochastic Partial Differential Equations Using R and INLA. doi:`10.1201/9780429031892`.
